# Targeting phosphorylation circuits on CREB and CRTCs as the strategy to prevent acquired skin hyperpigmentation

**DOI:** 10.7150/ijbs.86536

**Published:** 2024-01-01

**Authors:** Song-Hee Kim, Changseon Na, Cheng-Yong Yun, Jun Gu Kim, Seung Tae Baek, Hyun Jin An, Jae Duk Lee, Seung Wha Lee, Jae-Kyung Jung, Bang Yeon Hwang, Sang-Bae Han, Youngoo Kim

**Affiliations:** 1 College of Pharmacy, Chungbuk National University, Cheongju 28160, Korea.; 2 R&D Center, The Skin's Co. Ltd, Jecheon 27116, Korea.; 3 R&D Center, Yeomyung Biochem Co. Ltd, Cheongju 28172, Korea.

**Keywords:** Melanogenesis, UV-B, α-MSH, SIK inhibitor, Yaku A

## Abstract

**Background:** The cAMP response element-binding protein (CREB) and CREB-regulated transcription coactivators (CRTCs) cooperate in the transcriptional activation of microphthalmia-associated transcription factor subtype M (MITF-M) that is a master regulator in the biogenesis, pigmentation and transfer of melanosomes at epidermal melanocytes. Here, we propose the targeting of phosphorylation circuits on CREB and CRTCs in the expression of MITF-M as the rationale to prevent skin hyperpigmentation by elucidating the inhibitory activity and mechanism of yakuchinone A (Yaku A) on facultative melanogenesis.

**Methods:** We employed human epidermal melanocyte cell, mouse skin, and mouse melanoma cell, and applied Western blotting, reverse transcription-polymerase chain reaction, immunoprecipitation and confocal microscopy to conduct this study.

**Results:** This study suggested that α-melanocyte stimulating hormone (α-MSH)-induced melanogenic programs could switch on the axis of protein kinase A-salt inducible kinases (PKA-SIKs) rather than that of PKA-AMP activated protein kinase (PKA-AMPK) during the dephosphorylation of CRTCs in the expression of MITF-M. SIK inhibitors rather than AMPK inhibitors stimulated melanin production in melanocyte cultures in the absence of extracellular melanogenic stimuli, wherein SIK inhibitors increased the dephosphorylation of CRTCs but bypassed the phosphorylation of CREB for the expression of MITF-M. Treatment with Yaku A prevented ultraviolet B (UV-B)-irradiated skin hyperpigmentation in mice and inhibited melanin production in α-MSH- or SIK inhibitor-activated melanocyte cultures. Mechanistically, Yaku A suppressed the expression of MITF-M via dually targeting the i) cAMP-dependent dissociation of PKA holoenzyme at the upstream from PKA-catalyzed phosphorylation of CREB coupled with PKA-SIKs axis-mediated dephosphorylation of CRTCs in α-MSH-induced melanogenic programs, and ii) nuclear import of CRTCs after SIK inhibitor-induced dephosphorylation of CRTCs.

**Conclusions:** Taken together, the targeting phosphorylation circuits on CREB and CRTCs in the expression of MITF-M could be a suitable strategy to prevent pigmentary disorders in the skin.

## Introduction

Ultraviolet (UV) radiation under sunlight can cause pigmentation, aging and carcinogenesis in the skin 1, 2. UV-A radiation stimulates immediate pigmentation depending on the oxidative modification of existing melanin pigments and spatial redistribution of existing melanosomes to the epidermal upper layers but is independent of *de novo* synthesis of melanin pigments 1, 3. UV-B radiation leads to delayed pigmentation from the increased production of heavily pigmented melanosomes at epidermal melanocytes and up-regulation of melanogenic hormones at epidermal keratinocytes 1, 4. UV-B radiation stabilizes and activates p53, a transcription factor that up-regulates the expression of pro-opiomelanocortin (POMC) 1, 5. α-Melanocyte stimulating hormone (α-MSH) is generated from POMC through post-translational processing, and secreted from epidermal keratinocytes 6. In addition to α-MSH, the p53 transcription factor also occupies on the promoter region of other melanogenic hormones for maximal induction at epidermal keratinocytes, thus facilitating pigmentation in the skin 1, 5.

**Figure 1:**
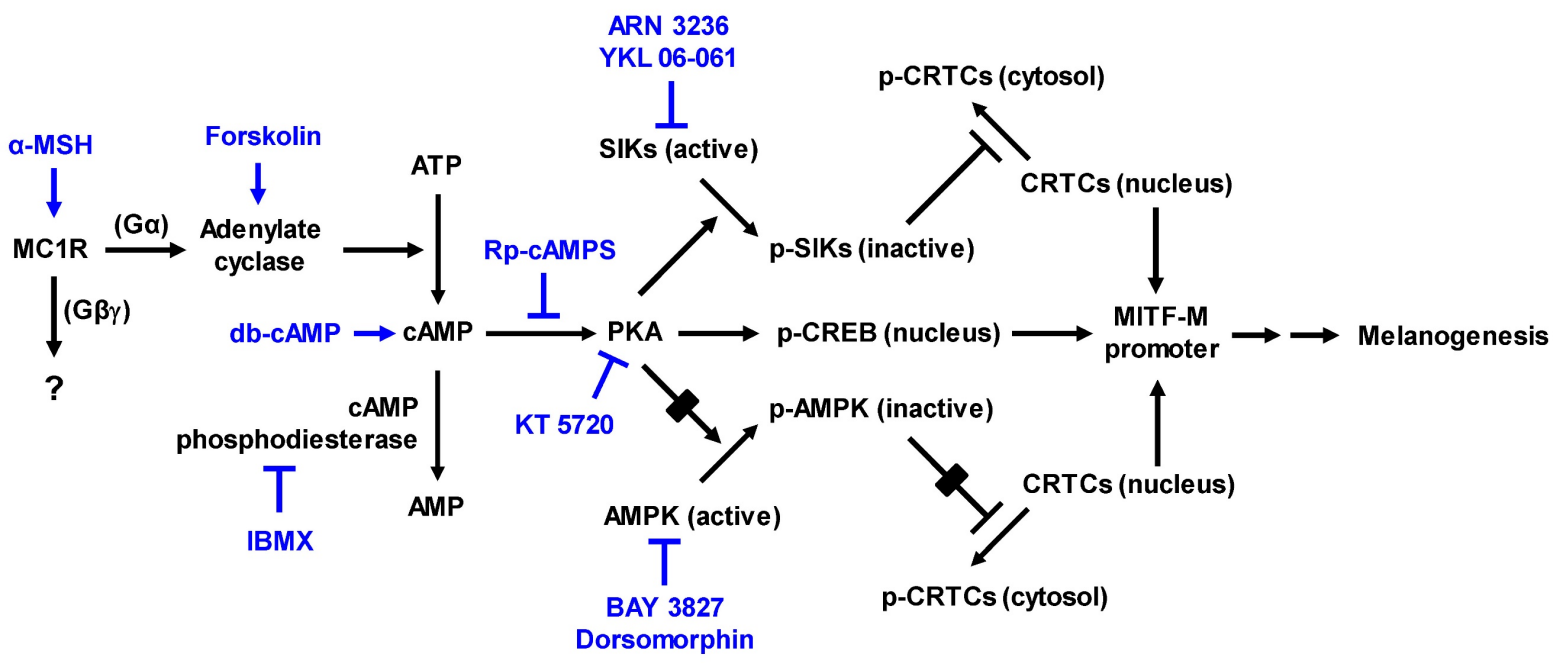
** Proposed signal transducers in α-MSH-induced and cAMP-dependent melanin production of melanocytes through phosphorylation circuits on CREB and CRTCs in the expression of MITF-M.** Pharmacological agents, such as activators or inhibitors of melanogenic signaling, were employed and are highlighted as blue-colored words. In the current study, HEM and B16F0 cells stimulated melanin production in the presence of SIK inhibitors rather than AMPK inhibitors. B16F0 cells switched on the axis of PKA-SIKs rather than that of PKA-AMPK in response to db-cAMP.

As shown in Figure 1, α-MSH specifically interacts with the melanocortin 1 receptor (MC1R) on the surface of epidermal melanocytes in the skin, which results in the increase of cyclic adenosine monophosphate (cAMP) levels by allosteric activation of adenylate cyclase through Gα subunit 7-9. In turn, cAMP binds to the regulatory subunits in the inactive holoenzyme of protein kinase A (PKA), and dissociates the catalytic subunits from the regulatory subunits, thus activating the kinase activity of PKA on cellular substrates, including cAMP response element (CRE)-binding protein (CREB), salt-inducible kinases (SIKs), and AMP-activated protein kinase (AMPK) 8-11. PKA directly phosphorylates nuclear CREB, a transcription factor that interacts with the *cis*-acting CRE motif on promoter region of microphthalmia-associated transcription factor subtype M (MITF-M), which plays an important role in the recruitment of CREB-binding protein (CBP) and p300 8, 12, 13. CBP and p300 (CBP/p300) show acetyltransferase activity on nucleosomal histones to become transcriptionally active in the chromatin remodeling 12, 13. As shown in Figure 1, current study proposed that α-MSH could switch on the axis of PKA-SIKs rather than that of PKA-AMPK during the activation of CREB-regulated transcription coactivators (CRTCs) in the cAMP-dependent expression of MITF-M of mouse melanoma B16F0 cells. Structurally unrelated SIK inhibitors (ARN 3236 and YKL 06-061) rather than AMPK inhibitors (BAY 3827 and dorsomorphin) could stimulate melanin production in the cultures of human epidermal melanocyte (HEM) or B16F0 cells, wherein SIK inhibitors up-regulated the expression of MITF-M through the dephosphorylation of CRTCs and not through the phosphorylation of CREB. After SIKs-added phosphor (p) groups are removed by calcineurin, CRTCs translocate from the cytosol to the nucleus, and cooperate with CREB in the nucleus to recruit the transcription machinery onto the promoter region of MITF-M for transcriptional activation 10, 14-16.

MITF-M can be inducible under facultative melanogenesis in melanocytes, and plays an important role as the master transcription factor that interacts with *cis*-acting M-box and E-box in the promoter regions of MITF-M-target genes, encoding melanogenic proteins for the biogenesis, pigmentation and transfer of melanosomes 17-19. Melanogenic programs synthesize melanin pigments, such as eumelanin (black-brown) and pheomelanin (yellow-red), in the melanosomes of melanocytes at the epidermal-dermal border 20. Tyrosinase (TYR) has dual catalytic activities of tyrosine hydroxylase and dopa oxidase, converting tyrosine to dopaquinone, in the biosynthetic pathways of eumelanin and pheomelanin 20, 21. TYR-related protein 1 (TRP-1) and dopachrome tautomerase (DCT) are also required in the biosynthesis of eumelanin but not that of pheomelanin 20, 21. Pigmented melanosomes are then transferred to the cytosol of keratinocytes in the overlaying epidermis, thus determining the skin color and shielding the nuclei of skin cells for protection against UV radiation-induced risks 1, 22.

**Figure 2:**
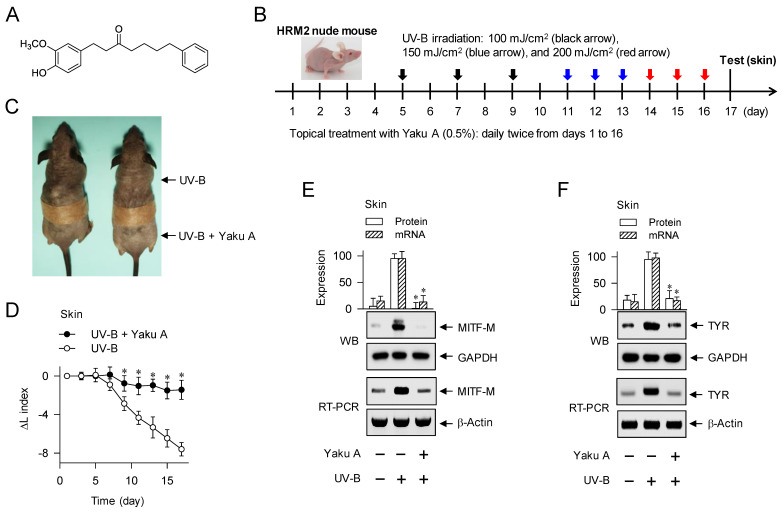

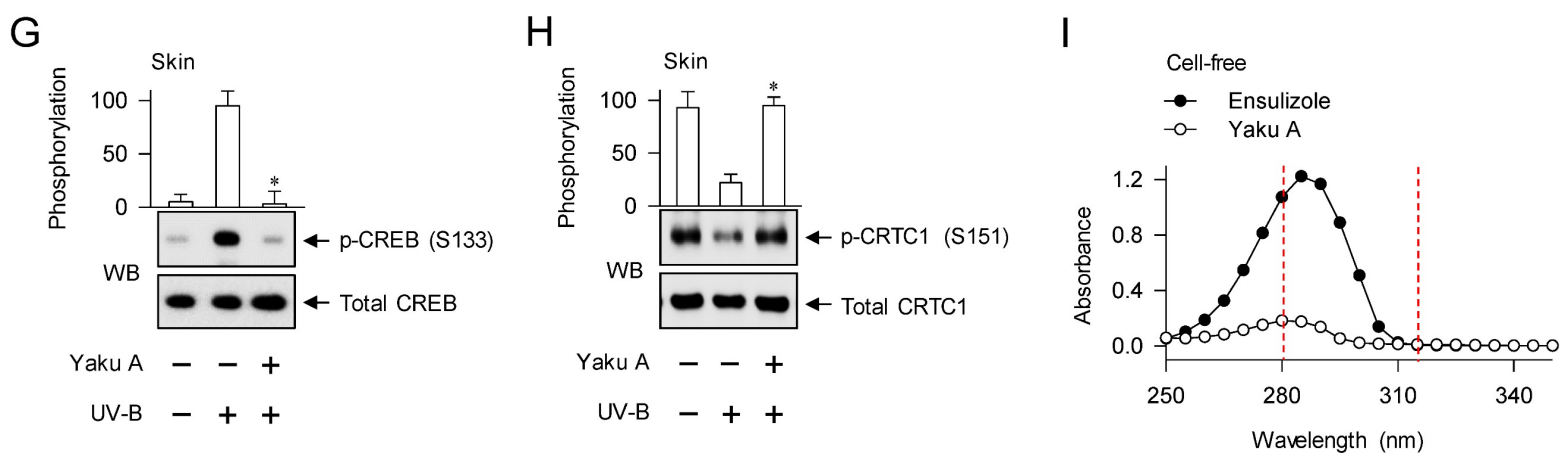
** Effect of Yaku A on UV-B-irradiated skin hyperpigmentation in mice.** (A) Chemical structure of Yaku A. (B) Experimental protocol of hyperpigmentation in the skin. The dorsal skins of HRM2 mice were topically treated with Yaku A in daily twice, and irradiated with increasing doses of UV-B at the time points indicated by arrows. The skin was biopsied at day 17. (C) Yaku A inhibited visual hyperpigmentation at day 17. (D) The lightening index was measured once per every 2 days. Yaku A restored the change of lightening (ΔL) index in UV-B-irradiated skins to their normal status. (E, F) Yaku A suppressed UV-B-induced expression of MITF-M or TYR in the biopsied skin. (G, H) Yaku A inhibited UV-B-induced phosphorylation of CREB at the S133 residue and dephosphorylation of CRTC1 at the S151 residue in the biopsied skin. (I) Yaku A did not absorb UV-B light. Three independent tests with 3 to 4 mice per group. *P < 0.05 vs. UV-B alone.

Excess production and aberrant distribution of heavily pigmented eumelanin are responsible for pigmentary disorders in the skin, such as melasma, freckles and senile lentigo 23. In the current study, we propose the targeting phosphorylation circuits on CREB and CRTCs in the expression of MITF-M as antimelanogenic rationales by elucidating the inhibitory activity and mechanism of yakuchinone A (Yaku A, Figure 2A) on facultative melanogenesis. *Alpinia oxyphylla*, a plant of the Zingiberaceae family, and its constituent Yaku A inhibit the catalytic activity of mushroom TYR 24, expecting similar effects on mammalian TYRs that are involved in the production of melanin pigments. In the preliminary experiments, fruits of *A. oxyphylla* were extracted with 70% ethyl alcohol (EtOH), and defined as ID 36353 according to the international nomenclature of cosmetics ingredient (INCI). The extracts of *A. oxyphylla* inhibited α-MSH-induced melanin production in B16F0 cells, as did arbutin (Figure S1A and B). Arbutin is a skin whitener approved by Korea food and drug administration (FDA), and employed as a positive control agent in this study. Moreover, Yaku A was isolated from the extracts of *A. oxyphylla* (Appendix S1; Figure S2). In the current study, we focused on the antimelanogenic activity of Yaku A *in vivo* and its mechanism of action. Topical treatment with Yaku A protected the mice from UV-B-irradiated hyperpigmentation in the skin. Moreover, Yaku A inhibited melanin production in α-MSH- or SIK inhibitor-activated melanocyte cultures. As the molecular bases, Yaku A suppressed the expression of MITF-M via dually targeting the i) cAMP-dependent dissociation (activation) of inactive PKA holoenzyme in α-MSH-induced phosphorylation of CREB coupled with dephosphorylation of CRTCs, and ii) translocation of CRTCs from the cytosol to the nucleus after SIK inhibitor-induced dephosphorylation of CRTCs independent of cAMP. Based on these antimelanogenic results, we propose the targeting phosphorylation circuits on CREB and CRTCs in the expression of MITF-M as a suitable strategy to prevent pigmentary disorders in the skin.

## Materials and methods

### Yaku A from *A. oxyphylla*

Yaku A (purity, >97%) was isolated from extracts of *A. oxyphylla* (Appendix S1; Figure S2). Spectral data of Yaku A were UV (MeOH) λ_max_ 280 nm; IR ν_max_ (film) 3560, 1710, 1275 cm^-1^; ^1^H NMR (400 MHz, CDCl_3_) *δ*_H_ 1.60 (4H, m, H-5, 6), 2.39 (2H, t, *J* = 7.0 Hz, H-4), 2.59 (2H, t, *J* = 7.0 Hz, H-7), 2.67 (2H, t, *J* = 7.5 Hz, H-2), 2.81 (2H, t, *J* = 7.0 Hz, H-1), 3.85 (3H, s, 3′-OCH_3_), 5.50 (1H, br s, 4′-OH), 6.66 (1H, dd, *J* = 2.0, 8.0 Hz, H-6′), 6.70 (1H, d, *J* = 1.9, H-2′), 6.82 (1H, d, *J* = 8.0, H-5′), 7.13 - 7.19 (3H, m, H-2′′, 4′′, 6′′) 7.24 - 7.28 (2H, m, H-3′′, 5′′); ^13^C NMR (100 MHz, CDCl_3_) *δ*_C_ 23.4 (C-5), 29.5 (C-1), 30.9 (C-6), 35.7 (C-7), 42.9 (C-4), 44.7 (C-2), 55.9 (3′-OCH_3_), 111.0 (C-2′), 114.3 (C-5′), 120.7 (C-6′), 125.8 (C-4′′), 128.3 (C-2′′, 6′′), 128.4 (C-3′′, 5′′), 133.1 (C-1′), 142.2 (C-1′′), 143.9 (C-4′), 146.4 (C-3′), 210.3 (C-3); HRESIMS *m/z* 313.1804 [M + H]^+^ (calculated for C_20_H_25_O_3_, 313.1798).

### Pharmacological agents

Drugs and chemicals were α-MSH (Sigma-Aldrich, M4125) as melanogenic hormone; arbutin (Sigma-Aldrich, A4256) as skin whitener; ARN 3236 (Selleckchem, S8543) and YKL 06-061 (MedChemExpress, HY-120056) as SIK inhibitors; BAY 3827 (Selleckchem, S9833) and dorsomorphin (Sigma-Aldrich, 171260) as AMPK inhibitors; forskolin (Sigma-Aldrich, F6886) as adenylate cyclase activator; 3-isobutyl-1-methylxanthine (IBMX, Sigma-Aldrich, 410957) as cAMP phosphodiesterase inhibitor; KT 5720 (Sigma-Aldrich, K3761) as PKA inhibitor; *N*^6^,2'-*O*-dibutyryl-cAMP (db-cAMP, Sigma-Aldrich, D0627) as cAMP agonist; and Rp-adenosine 3',5'-cyclic monophosphorothioate (Rp-cAMPS, Sigma-Aldrich, A165) as cAMP antagonist.

### Skin hyperpigmentation by UV-B radiation

Dorsal skins of HRM2 nude mice (Central Lab Animals) were topically treated with Yaku A, dissolving in propylene glycol: EtOH: H_2_O (5: 3: 2) and applying each 1-ml treatment of 0.5% Yaku A per mouse skin, and irradiated with UV-B light according to the experimental protocol (Figure 2B). Lightening index was measured once per every 2 days in UV-B-exposed and pigmented skins using chromameter. Skin tissues were biopsied at day 17, and their protein extracts or total RNAs were prepared for Western blotting or RT-PCR analysis. Animal study (permission number, CBNUR-1253-19) was approved by the Animal Experimentation Ethics Committee of CBNU institute.

### Cell culture

Neonatal foreskins-originated HEM cells (Thermo Fischer Scientific, C1025C) were cultured in the medium 254 (Gibco, M254) containing melanocyte growth supplement (Gibco, S002). B16F0 cells (ATCC, CRL-6322) or mouse keratinocyte PAM212 cells 25 were cultured in the Dulbecco's modified Eagle's medium (DMEM, Sigma-Aldrich, D2902) supplemented with 10% fetal bovine serum (FBS, Coring, 35-01-CV) and antibiotic-antimycotic cocktail (Gibco, 15240062). For co-culture model, B16F0 cells (5 x 10^4^) were seeded on 6-well culture plates and pre-incubated for 24 h, and PAM212 cells (5 x 10^4^) on trans-well inserts in a pore size of 0.4 μm (Corning, 3412) and pre-incubated for 24 h. Trans-well inserts containing PAM212 cells were transferred onto the culture plates containing B16F0 cells, and cultured in DMEM supplemented with 10% FBS and antibiotic-antimycotic cocktail in an atmosphere of 37°C and 5% CO_2_.

### Melanin quantification

HEM or B16F0 cells were stimulated 100 nM α-MSH, 30 µM forskolin, 3 mM db-cAMP, 100 µM IBMX, 3 µM ARN 3236 or 1 µM YKL 06-061 for 72 h. The cells were disrupted in 0.9 N NaOH and 50% dimethyl sulfoxide (DMSO, Sigma-Aldrich, 34869) at 80°C for 2 h. Amounts of melanin pigments were determined by measuring absorbance values at wavelength 405 nm and represented as a relative fold.

### Western blot (WB) analysis

Protein extracts were prepared from skin tissuess, HEM or B16F0 cells, resolved on SDS-polyacrylamide gels by electrophoresis, and blotted to PVDF membranes (Merck-Millipore, IPVH00010). After blocking in 5% non-fat milk (BD, 232100) dissolved in Tris-buffered saline (TBS) containing Tween 20 (Biosesang, TR2007-100-74), blots were incubated with primary antibody at 4°C for 15 h followed by secondary antibody at 25°C for 1 h. Immune complexes on the gels were detected using chemiluminescence kit (Thermo Fischer Scientific, 34095; GE Healthcare, RPN2232). Primary antibodies were anti-AMPKα (Cell Signaling Technology, 2532); anti-CREB (Cell Signaling Technology, 9197); anti-CRTC1 (Cell Signaling Technology, 2587); anti-CRTC2 (Merck Millipore, ST1099); anti-CRTC3 (Santa Cruz Biotechnology, sc-390712); anti-MITF-M (Abcam, ab12039); anti-p-AMPKα (Cell Signaling Technology, 4185); anti-p-CREB (Cell Signaling Technology, 9198); anti-p-CRTC1 (Cell Signaling Technology, 3359); anti-p-SIK3 (Abcam, ab225633); anti-SIK3 (Abcam, ab227044); anti-TYR (Santa Cruz Biotechnology, sc-20035); anti-GAPDH (Cell Signaling Technology, 5174); and anti-histone H1 (Santa Cruz Biotechnology, sc-8030). Secondary antibodies were horseradish peroxidase (HRP)-labeled anti-mouse IgG (Thermo Fisher Scientific, 31430); and HRP-labeled anti-rabbit IgG (Thermo Fisher Scientific, 31460).

### RT-PCR analysis

Total RNAs were prepared from skin tissues and cell cultures of HEM or B16F0 using NucleoZOL (Macherey-Nagel, 740404), and subjected to RT-PCR analysis. Briefly, total RNAs were incubated with oligo-dT as a primer at 42°C for 1 h for reverse transcription. Single-stranded cDNAs were subjected to PCR for 28-30 cycles, in which one cycle consisted of 94°C for 30 sec (denaturation), 50-60°C for 2 min (annealing), and 72°C for 1 min (extension). Amplified transcripts as double-stranded cDNA were resolved on agarose gels by electrophoresis and visualized by staining with EcoDye (Biofact, ES301). Nucleotide sequences of RT-PCR primers were described in Table S1.

### Cell viability assay

B16F0, HEM or PAM212 cells were incubated with Yaku A for 72 h, and reacted with 1 mg/ml 3-(4,5-dimethylthiazol-2-yl)-2,5-diphenyltetrazolium bromide (MTT, Sigma-Aldrich, M5655) for another 30 min. Formazan crystals were dissolved in DMSO, and their absorbance values were measured at wavelength 590 nm.

### Immunoprecipitation (IP) assay

B16F0 cells were disrupted using a lysis buffer (50 mM Hepes pH 7.5, 0.1% NP-40, 5 mM EDTA, 150 mM NaCl, and 1 mM DTT) supplemented with protease inhibitor cocktail (GenDepot, P3100). Whole cell extracts were incubated with anti-PKA-RIIβ antibody (Santa Cruz Biotechnology, sc-376778) overnight at 4°C and then with protein G-sepharose beads (GE healthcare, 17-0618-01) at 4°C for another 2 h. These IP complexes were resolved on SDS-polyacrylamide gels by electrophoresis and probed with another antibody against anti-PKA-Cα (Cell Signaling Technology, 4782) to detect the co-precipitates.

### PKA activity assay

Lysates of B16F0 cells or catalytically active rhPKA (Sigma-Aldrich, 14-440) were reacted with 83 μM Kemptide (Promega, V5601) as substrate in the presence of 10 μCi [γ-^32^P]-ATP (Perkin Elmer, NEG002A) at 30°C for 30 min. The P81 phosphocellulose filter was spotted with a part of reaction mixtures, washed with 0.75% H_3_PO_4_ followed by acetone, and measured count per min (cpm) using scintillation counter.

### Confocal microscopy

B16F0 cells were mounted onto poly-Lys-coated glass slides and fixed with 4% *p*-formaldehyde. Cells on the glass slides were permeabilized with PBS containing 0.5% Triton X-100 (Biosesang, PR4007), and blocked in PBS containing 1% bovine serum albumin (Sigma-Aldrich, A9647). After washing with PBS, cells on the glass slides were reacted with anti-CREB, anti-p-CREB or anti-CRTC1 antibody overnight at 4°C and stained with Alexa Fluor 488-conjugated secondary antibody (Invitrogen, A-11034) at room temperature for 1 h. Cells on the glass slides were further incubated with 4',6-diamidino-2-phenylindole (DAPI, Vector Lab, H1200) to stain DNA in the nucleus, and examined under confocal microscope (Carl Zeiss LSM 980).

### Luciferase reporter assay

B16F0 cells were transfected with luciferase reporter construct, MITF-M-Luc or TYR-Luc, using lipofectamine kit (Invitrogen, 11668). *Renilla* control vector was co-transfected as a reference of transfection efficiency. Lysates of transfected cells were subjected to dual luciferase assays (Promega, E1910). Firefly luciferase activity, indicating the promoter activity of MITF-M or TYR, was normalized to *Renilla* luciferase activity.

### Chromatin immunoprecipitation (ChIP) assay

An experimental protocol was supplied with a pre-mix kit (Merck-Millipore, 17-295). Briefly, cellular DNA and proteins were cross-linked by incubation with 1% formaldehyde at 25°C for 10 min. Chromatin fragments with about 200-500 base pairs of DNA in size were prepared by sonication, incubated with anti-CRTC1, anti-CRTC2 or anti-CRTC3 antibody overnight at 4°C and precipitated with protein A-sepharose bead. The elutes were incubated with 80 mM NaCl at 65°C for 6 h followed by 0.5 mg/ml proteinase K (Sigma-Aldrich, P2308) and 10 mM EDTA at 45°C for 1 h. Input or ChIP DNAs were subjected to PCR encompassing CRE motif in the promoter of MITF-M. Nucleotide sequences of PCR primer were as follows; forward 5′-TGGGGACTTGGCCTTGATCT-3′ and reverse 5′-ATATCAGTTTCCCTGCTGGCT-3′.

### Dopa oxidation assay

Lysates of B16F0 cells or catalytically active rhTYR (Enzo, BML-SE535) were reacted with 1 mM dopa (Sigma-Aldrich, D9628) as substrate in 63 mM sodium phosphate buffer (pH 6.5). TYR activity, initial velocity of dopa oxidation, was measured by measuring absorbance values at wavelength 475 nm per min, and represented as dopa oxidation (nmol/min).

### Statistical analysis

Results are represented as mean ± SEM (n = 3, unless otherwise specified). Data were analyzed using ANOVA and Student's t-test. *P* < 0.05 was considered statistically significant.

## Results

### Prevention of UV-B-irradiated skin hyperpigmentation in mice by Yaku A

To examine the antimelanogenic activity of Yaku A *in vivo*, dorsal skins of HRM2 nude mice were topically treated with Yaku A, irradiated with UV-B light in multiple 9-times exposure, and biopsied the skin at day 17 (Figure 2B). Treatment with Yaku A protected the skin from visual hyperpigmentation in the absence of corrosion (Figure 2C) as well as restored the lightening (ΔL) index in UV-B-exposed and pigmented skins to their normal status (Figure 2D). The protein and mRNA levels of melanocyte-specific genes encoding MITF-M and TYR were up-regulated in UV-B-irradiated and pigmented skins, which were suppressed by treatment with Yaku A (Figure 2E and F). Notably, phosphorylation of CREB at the S133 residue and dephosphorylation of CRTC1 at the S151 residue were increased in UV-B-irradiated and pigmented skins (Figure 2G and H). Treatment with Yaku A restored p-CREB and p-CRTC1 levels to their normal status (Figure 2G and H). CREB and CRTCs are ubiquitously expressed in the skin, liver and many other tissues for adaptive transcriptional responses to cAMP-messengered hormone and metabolic signals 14. Epidermal melanocytes exclusively produce melanin pigments in the skin but their population is minor in terms of numerical abundance, although epidermal keratinocytes and dermal fibroblasts, major constituent cells, secret melanogenic hormones and other paracrine factors 1, 26. We then examined whether melanogenic cells in the skin could drive the phosphorylation circuits on CREB and CRTC1, employing a co-cultured model between PAM212 keratinocyte and B16F0 melanoma cells. Both PAM212 and B16F0 cells stimulated the phosphorylation of CREB and the dephosphorylation of CRTC1 in response to UV-B irradiation, which were inhibited by treatment with Yaku A (Figure S3). However, Yaku A did not absorb UV-B light at all in contrast to ensulizole, which is a sun blocker (Figure 2I). These results suggest that Yaku A could prevent hyperpigmentation in UV-B-irradiated skins of mice and might regulate the phosphorylation circuits on CREB and CRTC1 in melanogenic cells.

### Inhibition of facultative melanogenesis in melanocyte culture by Yaku A

**Figure 3:**
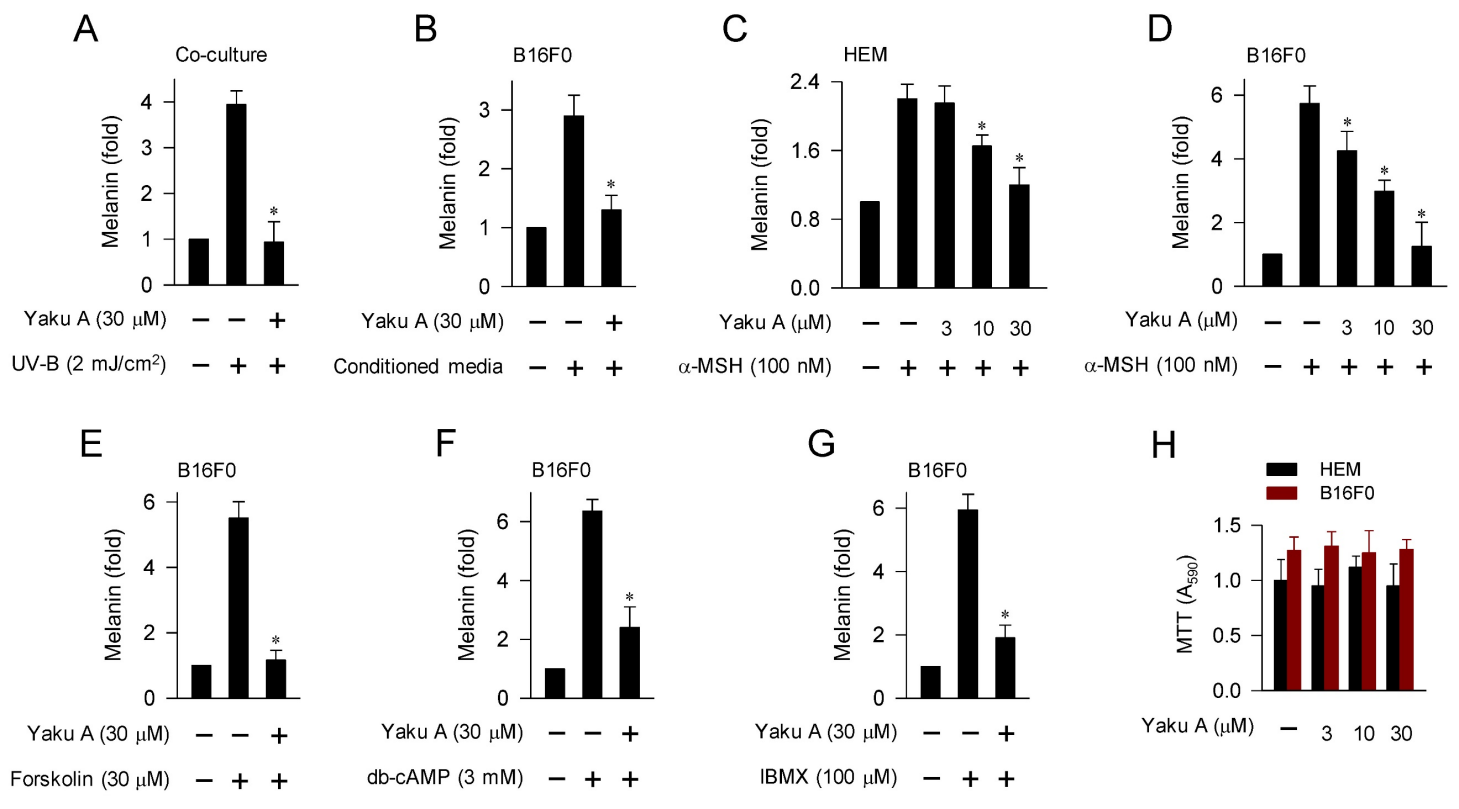
** Effect of Yaku A on melanin production in UV-B-, α-MSH- or cAMP elevator-activated cell cultures.** (A) Co-cultures of keratinocyte PAM212 cells and melanoma B16F0 cells were stimulated with a single irradiation of UV-B, and incubated for 72 h in the presence of Yaku A. Yaku A inhibited melanin production in UV-B-irradiated co-cultures. (B-G) Monocultures of B16F0 cells were incubated with melanogenic stimuli in the presence of Yaku A for 72 h. (B) Yaku A inhibited melanin production in conditioned media-activated B16F0 cells. Conditioned media were prepared from UV-B-irradiated PAM212 cultures. (C, D) Yaku A inhibited melanin production in α-MSH-activated HEM or B16F0 cells. (E-G) Yaku A inhibited melanin production in cAMP-elevated B16F0 cells. (H) Yaku A did not alter the viability of HEM or B16F0 cells during incubation for 72 h. *P < 0.05 vs. UV-B alone, conditioned media alone, α-MSH alone, forskolin alone, db-cAMP alone or IBMX alone.

UV-B irradiation triggers the production and secretion of melanogenic hormones, including α-MSH, from epidermal keratinocytes in the skin 1. In a paracrine fashion, α-MSH binds to its specific receptor MC1R at epidermal melanocytes and stimulates cAMP-dependent melanogenic programs 7, as shown in Figure 1. UV-B irradiation stimulated melanin production in a co-culture of keratinocyte PAM212 and melanoma B16F0 cells, which was inhibited by treatment with Yaku A (Figure 3A). Upon exposure to conditioned media from UV-B-irradiated PAM212 cultures, B16F0 cells stimulated melanin production, which was also inhibited by treatment with Yaku A (Figure 3B). Yaku A inhibited melanin production in α-MSH-activated HEM and B16F0 cells (Figure 3C and D), suggesting its antimelanogenic activity in both normal human melanocyte and mouse melanoma cell. Moreover, we examined whether Yaku A could affect melanin production in cAMP-elevated B16F0 cells. Yaku A inhibited forskolin-, db-cAMP-, or IBMX-induced melanin production (Figure 3E-G). Forskolin was employed as an activator of adenylated cyclase, db-cAMP as a cell-permeable cAMP agonist, and IBMX as an inhibitor of cAMP phosphodiesterase (Figure 1). However, the presence of Yaku A at concentrations exhibiting antimelanogenic activity did not alter the viability of B16F0, HEM or PAM212 cells (Figure 3H; Figure S4), apart from inducing non-specific cytotoxicity. These results suggest that Yaku A could inhibit UV-B-irradiated, α-MSH-induced and cAMP-dependent melanin production in cell-culture models.

### The axis of PKA-SIKs rather than that of PKA-AMPK was switched on in cAMP-dependent melanogenic programs, and the SIK inhibitor-induced melanin production was inhibited by Yaku A

PKA can up-regulate the expression of MITF-M through two mechanisms, phosphorylation of CREB coupled with dephosphorylation of CRTCs, in α-MSH-induced and cAMP-dependent melanogenic programs 8-10, as shown in Figure 1. PKA directly phosphorylates CREB to expand the transcriptional activity of CREB 8, 12, 13. PKA-catalyzed phosphorylation negatively regulates the catalytic activities of AMPK family of Ser/Thr kinases, such as SIKs and AMPK 10, 11. SIKs and AMPK directly phosphorylate CRTCs, which results in sequestering CRTCs in the cytosol by tethering with 14-3-3 protein 14. The inhibition of kinase activities in SIKs, either by PKA-catalyzed phosphorylation or SIK inhibitors (YKL 06-061 analogs), facilitates the dephosphorylation of CRTCs, thus leading to the translocation of CRTCs from the cytosol to the nucleus and ensuring that they interact with CREB on the promoter region of MITF-M 10, 14-16, 27.

**Figure 4:**
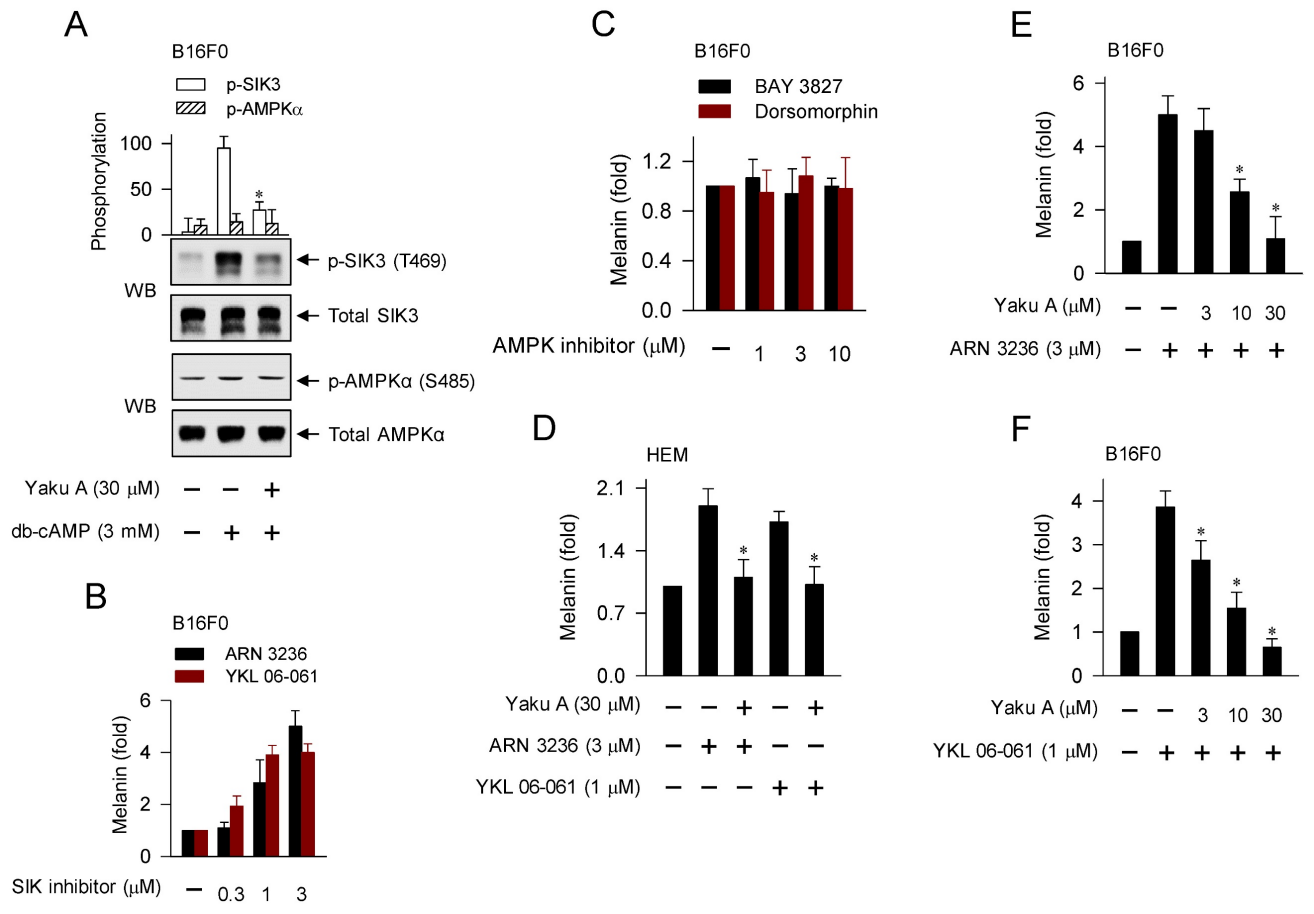
** Effects of Yaku A on the db-cAMP-dependent switching on PKA-SIKs axis and the SIK inhibitor-induced melanin production.** (A) B16F0 cells were pretreated with Yaku A for 2 h and stimulated with db-cAMP for 30 min in the presence of Yaku A. db-cAMP increased PKA-catalyzed phosphorylation of SIK3 at the T469 residue, which was inhibited by treatment with Yaku A. Either stimulation with db-cAMP or treatment with Yaku A did not alter PKA-catalyzed phosphorylation of AMPK (α subunit) at the S485 residue. (B, C) B16F0 cells were incubated with SIK inhibitors (ARN 3236 and YKL 06-061) or AMPK inhibitors (BAY 3827 and dorsomorphin) for 72 h. SIK inhibitors increased melanin production in the absence of extracellular melanogenic stimuli. AMPK inhibitors did not alter melanin production. (D-F) HEM or B16F0 cells were stimulated with SIK inhibitor in the presence of Yaku A for 72 h. Yaku A decreased SIK inhibitor-induced melanin production. *P < 0.05 vs. db-cAMP alone, ARN 3236 alone or YKL 06-061 alone.

Here, we examined whether cAMP-dependent melanogenic programs could switch on the axis of PKA-SIKs and/or PKA-AMPK during the dephosphorylation of CRTCs in the expression of MITF-M. B16F0 cells increased PKA-catalyzed phosphorylation of SIK3 at the T469 residue in response to db-cAMP, which was inhibited by treatment with Yaku A (Figure 4A). In contrast, either stimulation with db-cAMP or treatment with Yaku A did not alter PKA-catalyzed phosphorylation of AMPK (α-subunit) at the S485 residue in the cells (Figure 4A). Moreover, SIK inhibitors (ARN 3236 and YKL 06-061) stimulated melanin production in the absence of extracellular melanogenic stimuli (Figure 4B). AMPK inhibitors (BAY 3827 and dorsomorphin) did not alter melanin production in B16F0 or HEM cells (Figure 4C; Figure S5), suggesting that this might be a common phenomenon of melanocytes. For the first time, ARN 3236, structurally unrelated to YKL 06-061, could stimulate melanin production, but AMPK inhibitors did not alter melanin production. Notably, treatment with Yaku A inhibited melanin production in SIK inhibitor-activated HEM or B16F0 cells (Figure 4D-F). These results suggest that the axis of PKA-SIKs and the kinase activity of SIKs could play important roles in the dephosphorylation of CRTCs, thus regulating the expression of MITF-M in α-MSH-induced melanogenic programs, as proposed in Figure 1.

YKL 06-061 inhibits all isoforms of SIKs but does not affect any other AMPK-related kinases by targeting the gatekeeper Thr residue within a small hydrophobic channel to the ATP-binding site 10. Topical treatment with YKL 06-061 stimulates melanin pigmentation in human skin explants 27. ARN 3236 inhibits SIK isoforms with more efficiency than YKL 06-061 10. BAY 3827 and dorsomorphin inhibit the kinase activity of AMPK in a competitive mechanism with respect to ATP-binding site 28.

### Targeting cAMP-dependent dissociation of PKA holoenzyme in α-MSH-induced melanogenic programs by Yaku A

**Figure 5:**
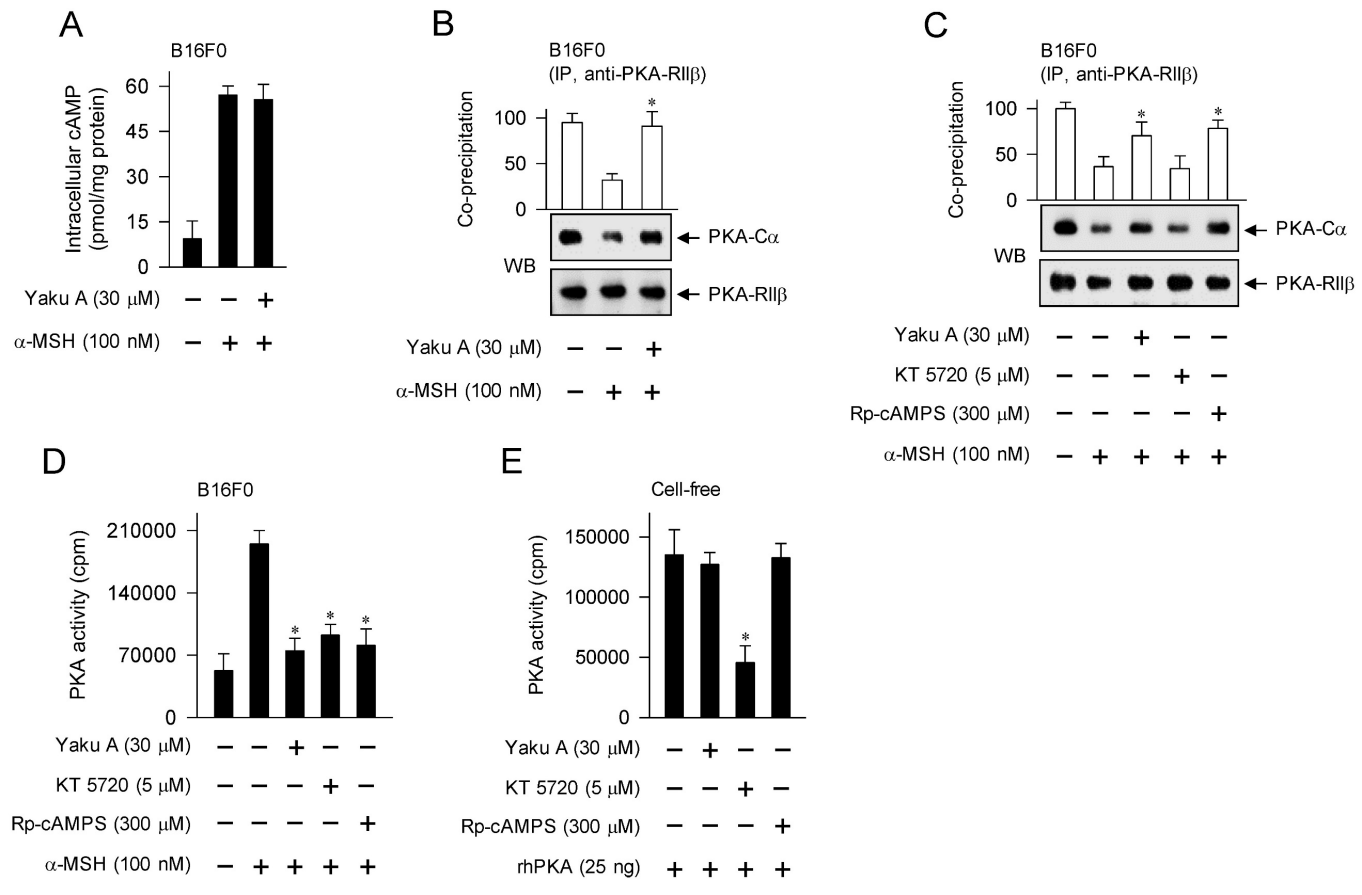
** Effect of Yaku A on the dissociation of PKA holoenzyme in α-MSH-induced melanogenic programs.** B16F0 cells were pretreated with Yaku A for 2 h and stimulated with α-MSH for 30 min in the presence of Yaku A. (A) Yaku A did not alter α-MSH-induced cAMP levels. (B, C). Whole-cell extracts were incubated overnight with anti-PKA-RIIβ for immunoprecipitation (IP) and probed with another antibody, anti-PKA-Cα. Yaku A inhibited α-MSH-induced dissociation of the catalytic subunit PKA-Cα from the regulatory subunit PKA-RIIβ. (D) Yaku A inhibited α-MSH-induced kinase activity of PKA in the cells. (E) rhPKA was incubated with Yaku A for 30 min and its kinase activity was determined. Yaku A did not alter catalytic activity of rhPKA in cell-free reactions. *P < 0.05 vs. α-MSH alone or rhPKA alone.

Yaku A inhibited α-MSH- or cAMP elevator-induced melanin production in melanocyte cultures (Figure 3C-G). To elucidate the antimelanogenic mechanism by Yaku A, we determined the cAMP levels using ELISA. Upon exposure to α-MSH, B16F0 cells increased the intracellular cAMP levels, which was unaffected by treatment with Yaku A (Figure 5A). Therefore, either the interaction of α-MSH with MC1R or the metabolic enzymes of cAMP might be excluded from the on-targets of Yaku A.

Subsequently, we tested the effects of Yaku A on cAMP-dependent proximal signaling events. B16F0 cells were pretreated with Yaku A for 2 h and stimulated with α-MSH for 30 min in the presence of Yaku A. Protein extracts were incubated with anti-PKA-RIIβ antibody for immunoprecipitation (IP), and then probed with another antibody against anti-PKA-Cα to detect the co-precipitates. Upon exposure to α-MSH, B16F0 cells dissociated the catalytic subunit of PKA (PKA-Cα) from the regulatory subunit of PKA (PKA-RIIβ), which was inhibited by treatment with YakuA (Figure 5B and C). Treatment with Rp-cAMPS but not KT 5720 inhibited α-MSH-induced dissociation of PKA holoenzyme in the cells, as did that with Yaku A (Figure 5C). KT 5720 was employed as a kinase inhibitor of the catalytic subunit that is already dissociated from PKA holoenzyme, and Rp-cAMPS as a cell-permeable cAMP antagonist that blocks the dissociation of PKA holoenzyme (Figure 1).

Furthermore, we evaluated whether the release of catalytic subunit from the inactive PKA holoenzyme could be correlated to the activation of kinase activity. Upon exposure to α-MSH, B16F0 cells increased the kinase activity of PKA (Figure 5D). Treatment with Yaku A inhibited α-MSH-induced PKA activity in the cells, as did that with KT 5720 or Rp-cAMPS (Figure 5D). To understand whether Yaku A could directly attack the catalytic subunit of PKA, rhPKA with the catalytic subunit alone was treated with Yaku A in cell-free reactions, and its kinase activity was measured. Treatment with Yaku A or Rp-cAMPS had no effects on the rhPKA-catalyzed kinase activity, whereas that with KT 5720 inhibited (Figure 5E). These results suggest that Yaku A could primarily inhibit the cAMP-dependent dissociation (activation) of inactive PKA holoenzyme in α-MSH-induced melanogenic programs.

### Regulation of CREB phosphorylation coupled with CRTC1 dephosphorylation in α-MSH-induced expression of MITF-M by Yaku A

**Figure 6:**
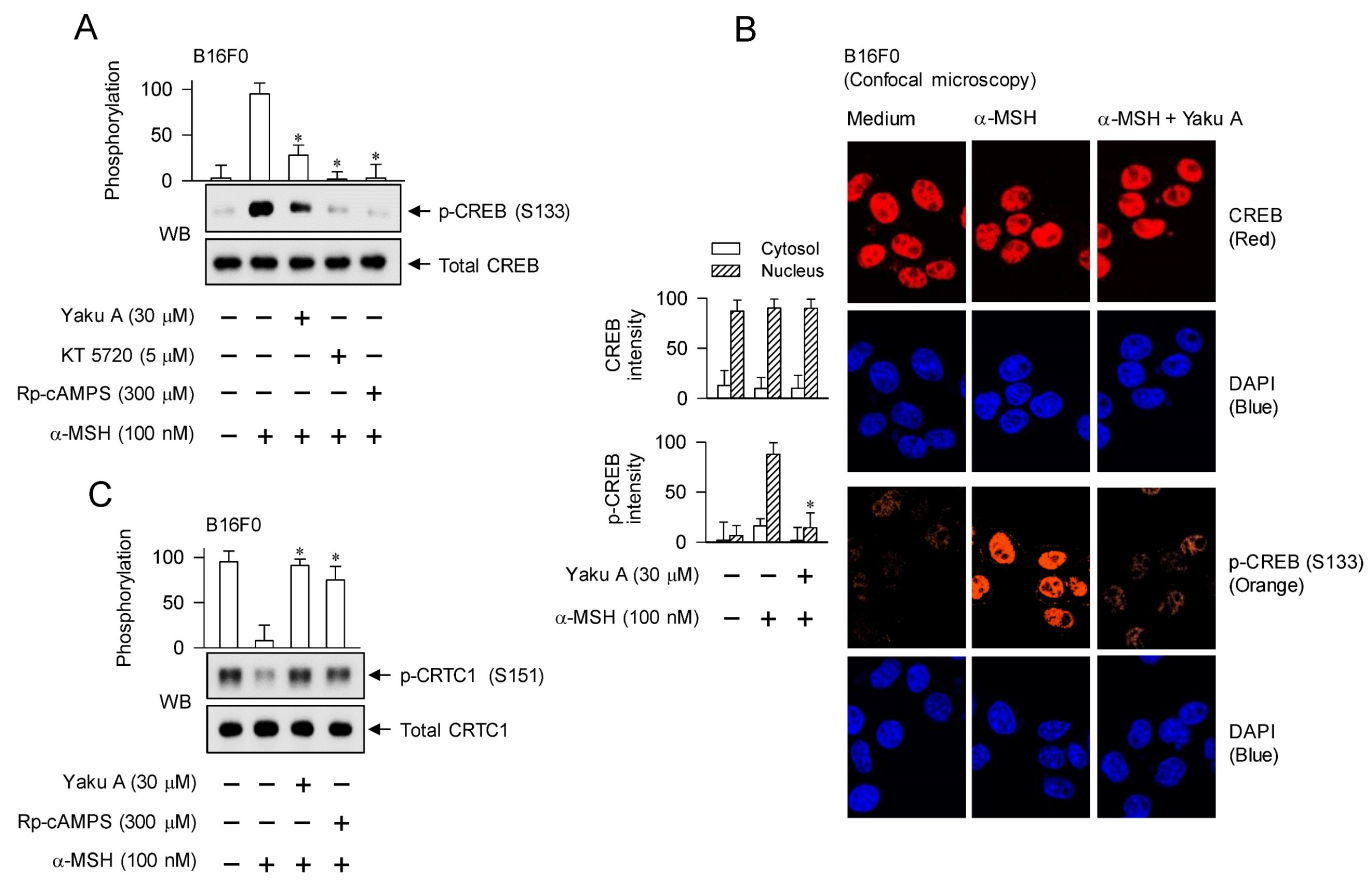

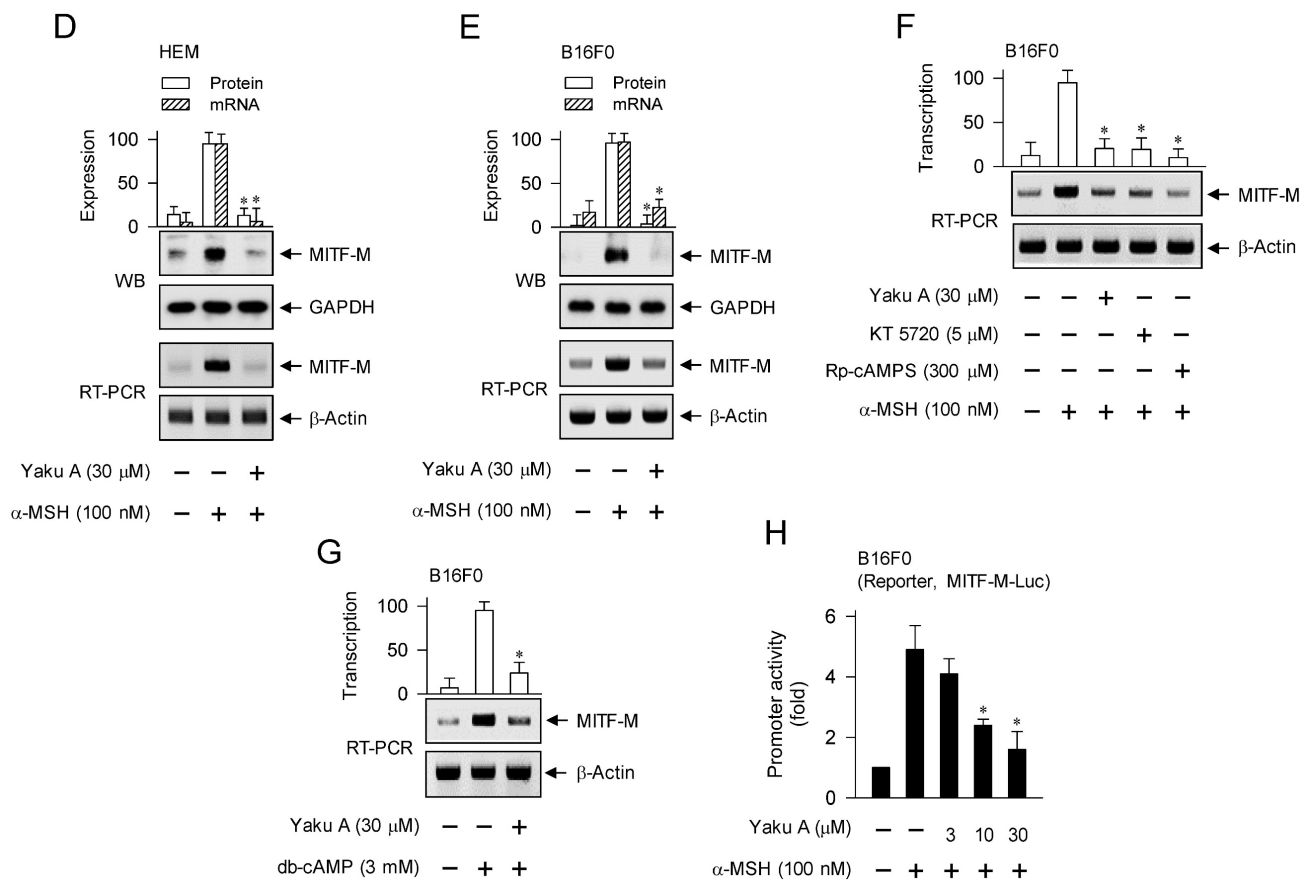
** Effects of Yaku A on the phosphorylation of CREB, the dephosphorylation of CRTC1 and the expression of MITF-M in α-MSH-induced melanogenic programs.** B16F0 or HEM cells were pretreated with Yaku A for 2 h and stimulated with α-MSH or db-cAMP for 30 min (A, C), 1 h (B), 2 h (mRNA levels in D-G) or 4 h (protein levels in D, E) in the presence of Yaku A. (A) Yaku A inhibited α-MSH-induced phosphorylation of CREB at the S133 residue. (B) CREB was localized in the nucleus of B16F0 cells, and its phosphorylation was occurred in the nucleus upon exposure to α-MSH. (C) Yaku A inhibited α-MSH-induced dephosphorylation of CRTC1 at the S151 residue. (D, E) Yaku A suppressed α-MSH-induced expression of MITF-M. (F) Treatment with KT 5720 or Rp-cAMPS suppressed α-MSH-induced transcription of MITF-M, as did that with Yaku A. (G) Yaku A suppressed db-cAMP-induced transcription of MITF-M. (H) B16F0 cells were transfected with MITF-M-Luc reporter construct and stimulated with α-MSH in the presence of Yaku A for 20 h. Yaku A inhibited α-MSH-induced promoter activity of MITF-M. *P < 0.05 vs. α-MSH alone or db-cAMP alone.

Phosphorylation circuits on CREB and CRTCs are essentially involved in the maximal induction of MITF-M at the promoter level in α-MSH-activated melanocytes 4, 18, 29, as shown in Figure 1. α-MSH-induced phosphorylation of CREB at the S133 residue was peaked at 30 min and then returned to low levels from 1 h to 4 h (Figure S6). However, α-MSH dephosphorylated CRTC1 at the S151 residue within 30 min and low levels of p-CRTC1 were sustained until 4 h (Figure S6). We then evaluated the effect of Yaku A on α-MSH-induced phosphorylation of CREB and dephosphorylation of CRTCs at the downstream position from PKA. Treatment with Yaku A inhibited PKA-catalyzed phosphorylation of CREB at the S133 residue in α-MSH-activated B16F0 cells, as did that with KT 5720 or Rp-cAMPS (Figure 6A). In the cellular dynamics using confocal microscopy, CREB was found in the nucleus of B16F0 cells (Figure 6B). Upon exposure to α-MSH, B16F0 cells increased PKA-catalyzed phosphorylation of CREB in the nucleus, which was inhibited by treatment with Yaku A (Figure 6B). Moreover, α-MSH increased the dephosphorylation of CRTC1 at the S151 residue in B16F0 cells (Figure 6C). Treatment with Yaku A restored p-CRTC1 levels to their normal status, as did that with Rp-cAMPS (Figure 6C). These results suggest that Yaku A could inhibit α-MSH-induced phosphorylation circuits on CREB and CRTC1, such as PKA-catalyzed phosphorylation of CREB coupled with PKA-SIKs axis-mediated dephosphorylation of CRTC1.

We then examined whether Yaku A could sequentially regulate α-MSH-induced expression of MITF-M at the downstream position from phosphorylation circuits on CREB and CRTCs. Upon exposure to α-MSH, HEM and B16F0 cells increased the protein and mRNA levels of MITF-M, which were suppressed by treatment with Yaku A (Figure 6D and E). The presence of KT 5720 or Rp-cAMPS in B16F0 cells also suppressed α-MSH-induced transcription of MITF-M, as did that of Yaku A (Figure 6F), suggesting that this transcription would be dependent of cAMP and PKA. Moreover, treatment with Yaku A suppressed db-cAMP-induced transcription of MITF-M (Figure 6G). The transcriptional regulation of MITF-M by Yaku A was further characterized using reporter assays. B16F0 cells were transfected with MITF-M-Luc, a luciferase reporter fused to the promoter region (-2200/+95) of MITF-M. Upon exposure to α-MSH, B16F0 cells harboring MITF-M-Luc increased the luciferase activity, indicating the promoter activity of MITF-M, which was inhibited by treatment with Yaku A (Figure 6H). Overall, Yaku A could primarily inhibit the cAMP-dependent dissociation of PKA holoenzyme, thus affecting α-MSH-induced melanogenic programs at the downstream positions, such as the phosphorylation circuits on CREB and CRTCs and the transcriptional activation of MITF-M.

### Targeting the nuclear import of CTRCs in SIK inhibitor-induced MITF-M expression and melanin production by Yaku A

**Figure 7:**
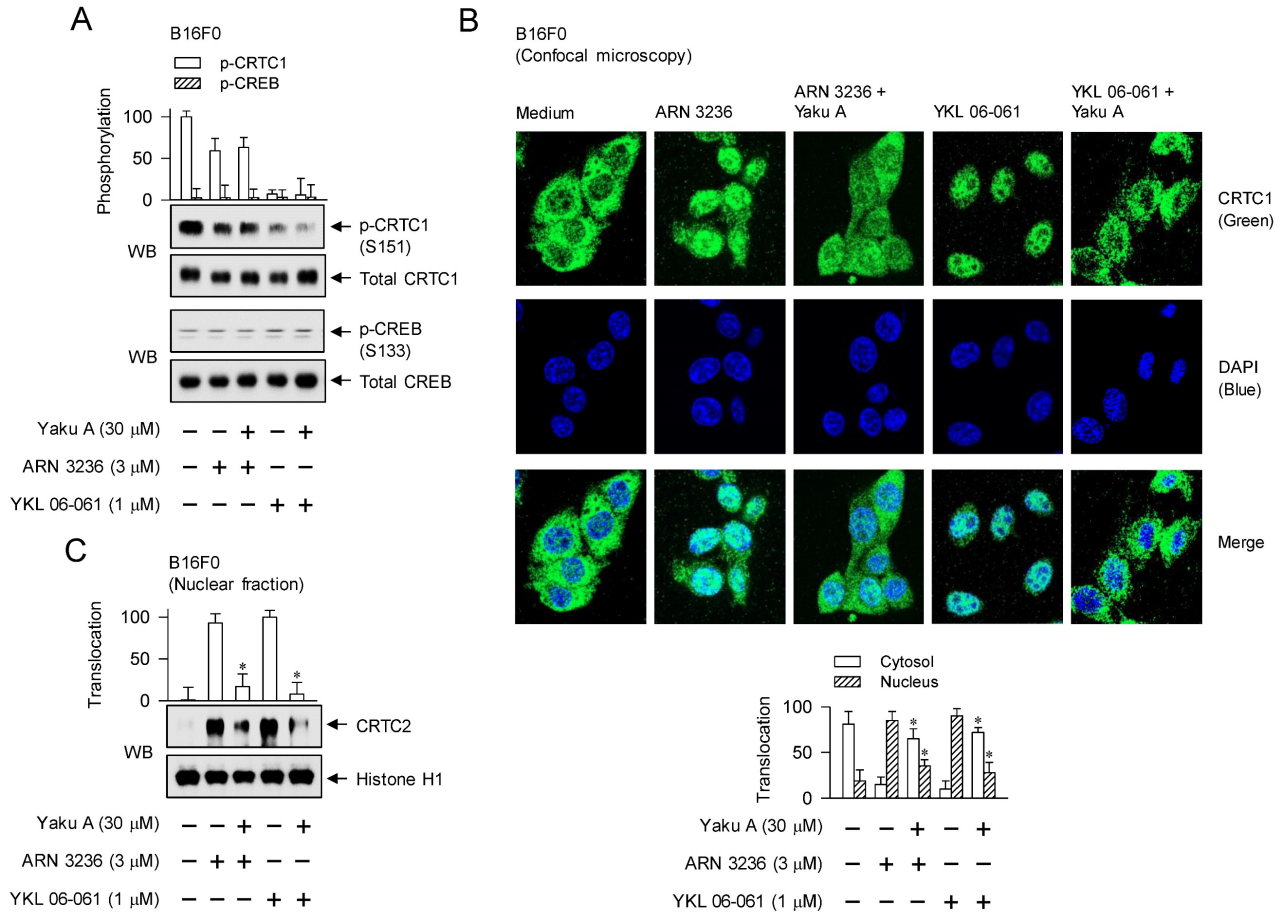

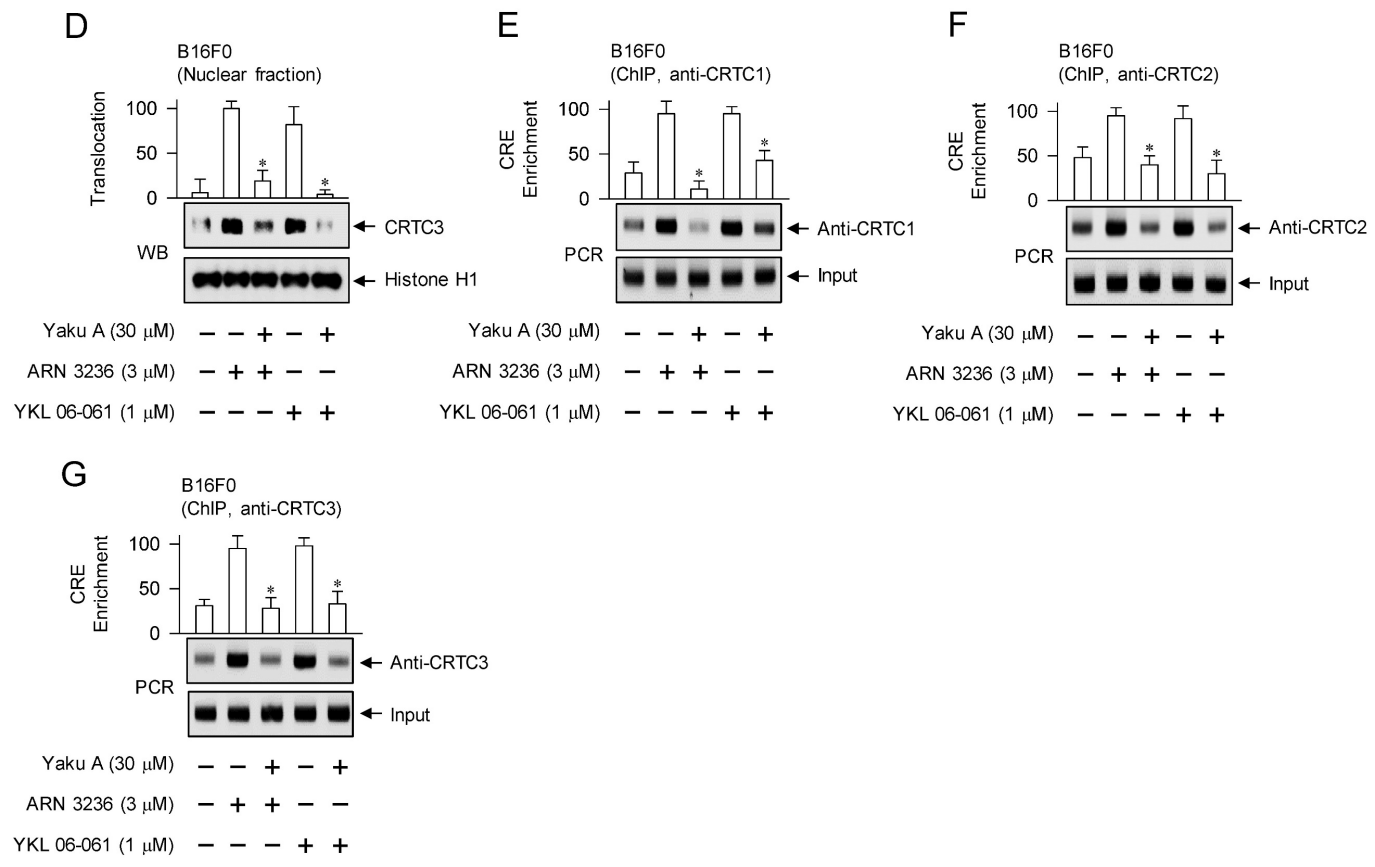
** Effects of Yaku A on the dephosphorylation of CRTC1, the nuclear import of CRTCs and the interaction of CRTCs with CREB in SIK inhibitor-induced melanogenic programs.** B16F0 cells were pretreated with Yaku A for 2 h and stimulated with SIK inhibitor (ARN 3236 or YKL 06-061) for 30 min (A), 1 h (B-D) or 90 min (E-G) in the presence of Yaku A. (A) SIK inhibitors decreased the dephosphorylation of CRTC1 at the S151 residue but bypassed the phosphorylation of CREB at the S133 residue, which were unaffected by treatment with Yaku A. (B) Yaku A blocked SIK inhibitor-induced nuclear import of CRTC1. CRTC1 was predominantly localized in the cytosol of B16F0 cell added with medium alone, and translocated from the cytosol to the nuclear upon exposure to SIK inhibitor. (C, D) Yaku A blocked SIK inhibitor-induced nuclear import of CRTC2 or CRTC3. (E-G) Yaku A inhibited the interaction of CRTC1, CRTC2 or CRTC3 with CREB on the CRE motif in promoter region of MITF-M. *P < 0.05 vs. ARN 3236 alone or YKL 06-061 alone.

Yaku A inhibited melanin production in SIK inhibitor (ARN 3236 or YKL 06-061)-activated melanocyte cultures (Figure 4D-F). SIK inhibitors increased the dephosphorylation of CRTC1 at the S151 residue, but did not phosphorylate CREB at the S133 residue in B16F0 cells (Figure 7A; Figure S7). Treatment with Yaku A had no effects on p-CRTC1 and p-CREB levels in SIK inhibitor-activated cells (Figure 7A). These results suggest that the kinase activity of SIKs would be required as a signal transducer at the upstream from phosphorylation circuits on CRTCs but not those on CREB, as shown in Figure 1.

Three isoforms of CRTCs (CRTC1, CRTC2 and CRTC3) are redundantly expressed in melanocytes, complementing in the expression of MITF-M, and have been reported as antimelanogenic targets at least in melanocyte cultures 30-33. CRTCs should be translocated from the cytosol into the nucleus to function as co-activators of transcription machinery, which essentially requires the removal of SIKs-added phosphate groups 10, 15. To elucidate the antimelanogenic mechanism by Yaku A, we examined the nuclear-cytoplasmic shuttling of CRTCs. Through confocal microscopy, we found that CRTC1 was predominantly located in the cytosol of B16F0 cells added with medium alone (Figure 7B). Upon exposure to SIK inhibitors, localization of CRTC1 shifted from the cytosol to the nucleus, which was inhibited by treatment with Yaku A (Figure 7B). Subsequently, we evaluated whether Yaku A could similarly affect the translocation of CRTC2 or CRTC3. Western blot (WB) analysis showed that exposure of SIK inhibitors increased the protein levels of CRTC2 or CRTC3 in the nucleus of B16F0 cells, wherein histone H1 was employed as a nucleus marker (Figure 7C and D). Treatment with Yaku A inhibited the nuclear import of CRTC2 or CRTC3 in SIK inhibitor-activated cells (Figure 7C and D).

The CREB-responsive CRE motif in the promoter region of MITF-M is located at -291/-284 34. CREB has different domain structures for occupying the CRE motif and interacting with CRTCs, but CRTCs cannot directly bind to the CRE motif 14. We conducted chromatin immunoprecipitation (ChIP) to determine whether the nuclear entry of CRTCs could be corrected with the binding ability to CREB on the CRE motif in promoter region of MITF-M. Chromatin fragments were precipitated using anti-CRTC1, anti-CRTC2 or anti-CRTC3 antibody, and subjected to PCR to determine the enrichment of the CRE motif in promoter region of MITF-M. Upon exposure to SIK inhibitors, B16F0 cells markedly enhanced the interaction between each isoform of CRTCs and CREB, which were inhibited by treatment with Yaku A (Figure 7E-G).

**Figure 8:**
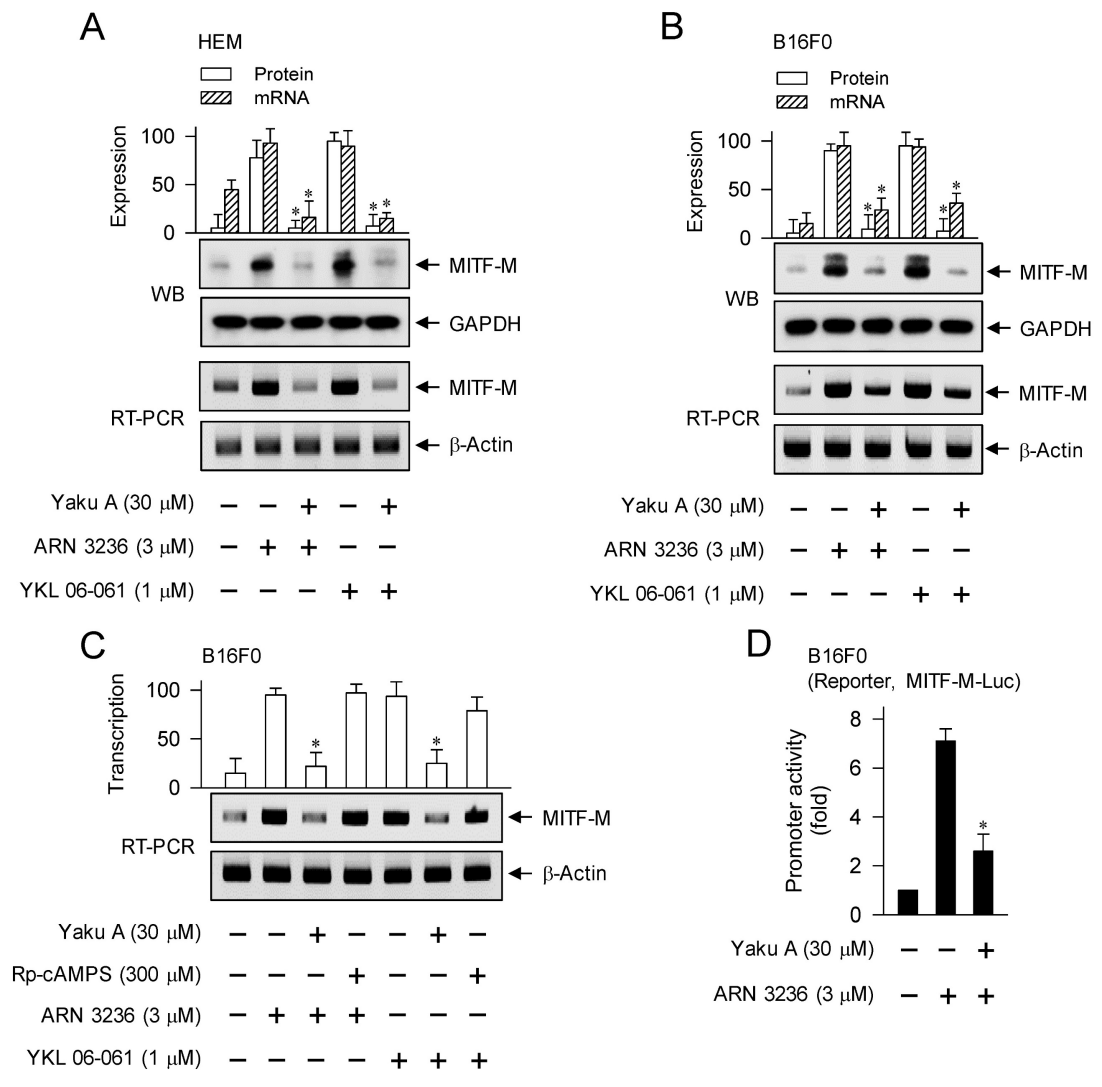
** Effect of Yaku A on the expression of MITF-M in SIK inhibitor-induced melanogenic programs.** HEM or B16F0 cells were pretreated with Yaku A for 2 h and stimulated with SIK inhibitor (ARN 3236 or YKL 06-061) for another 2 h (mRNA levels at A-C) or 4 h (protein levels at A, B) in the presence of Yaku A. (A, B) Yaku A suppressed SIK inhibitor-induced expression of MITF-M. (C) Treatment with Rp-cAMPS suppressed SIK inhibitor-induced transcription of MITF-M, as did that with Yaku A. (D) B16F0 cells were transfected with MITF-M-Luc reporter construct and stimulated with ARN 3236 in the presence of Yaku A for 20 h. Yaku A inhibited ARN 3236-induced promoter activity of MITF-M. *P < 0.05 vs. ARN 3236 alone or YKL 06-061 alone.

Upon exposure to SIK inhibitors, HEM and B16F0 cells increased the protein and mRNA levels of MITF-M, which were suppressed by treatment with Yaku A (Figure 8A and B). However, Rp-cAMPS did not alter SIK inhibitor-induced transcription of MITF-M (Figure 8C), suggesting that cAMP might not be involved as a signal transducer in SIK inhibitor-induced melanogenic programs. Moreover, treatment with Yaku A inhibited ARN 3236-induced promoter activity of MITF-M, wherein the reporter construct of MITF-M-Luc was transfected into B16F0 cells (Figure 8D). Overall, Yaku A could primarily block SIK inhibitor-induced nuclear import of CRTCs, and resultantly affect downstream melanogenic programs, such as the interaction of CRTCs with CREB on CRE motif and the transcriptional activation of MITF-M.

### Suppression of α-MSH- and SIK inhibitor-induced expression of MITF-M-target genes by Yaku A

MITF-M regulates the expression of TYR, TRP-1 and DCT as melanosomal enzymes during melanin biosynthesis, PMEL17 and Rab27A as non-enzymatic proteins during the biogenesis and transfer of melanosomes, and MC1R and EDNRB as receptors recognizing melanogenic hormones 17-19. MITF-M-responsive M-box and E-box, *cis*-acting DNA elements in the promoter regions of MITF-M-target melanogenic genes, are located at sites -107/-97 and -13/-6 in the TYR gene; at -211/-201 in the TRP-1 gene; at -137/-127 in the DCT gene; at +583/+591 in the PMEL17 gene; at -58/-51 and -46/-39 in the Rab27A gene; and at -479/-474 in the MC1R gene 18, 19, 35.

**Figure 9:**
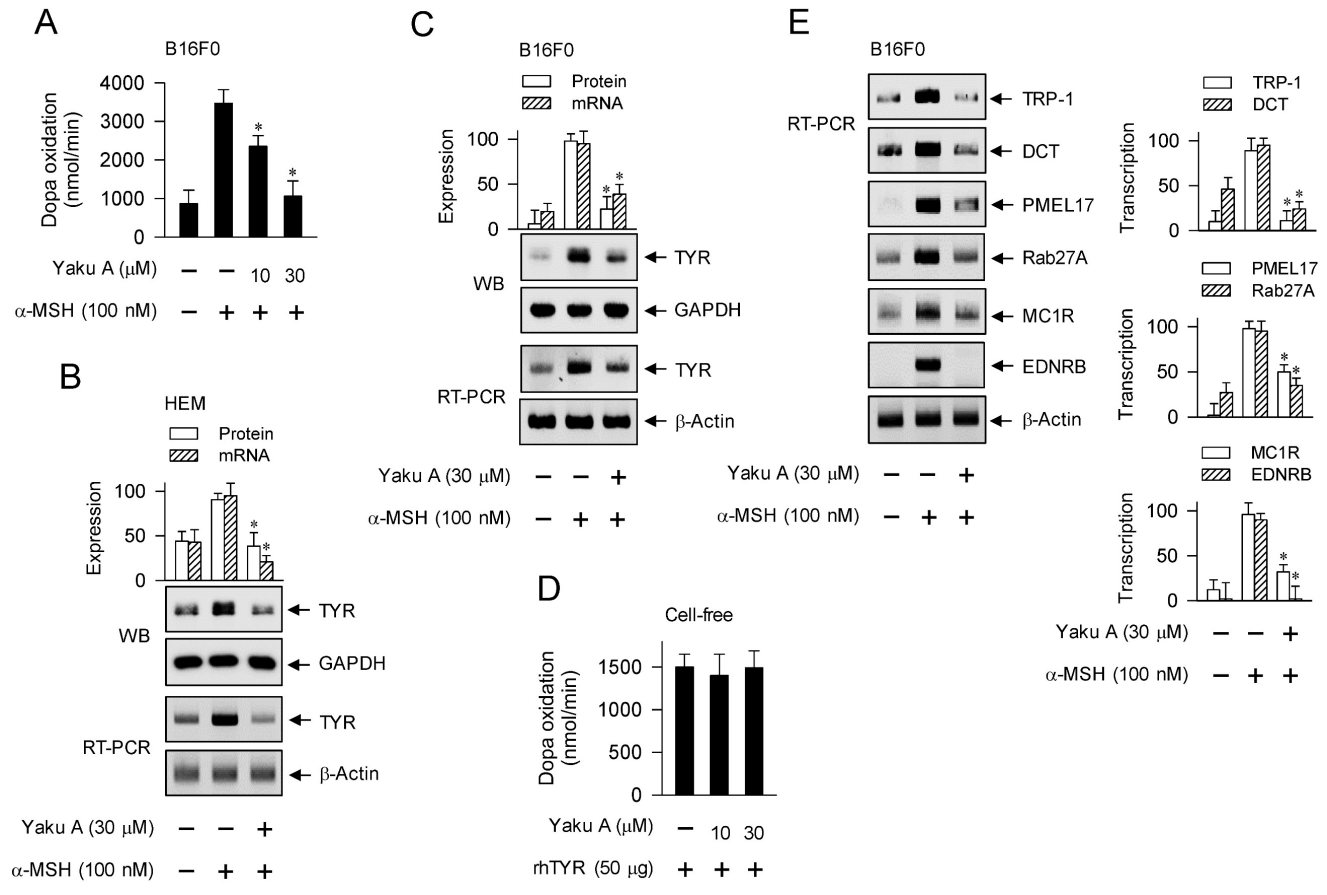
** Effects of Yaku A on the dopa oxidation activity of TYR and the expression of MITF-M-target genes in α-MSH-induced melanogenic programs.** HEM or B16F0 cells were pretreated with Yaku A for 2 h and stimulated with α-MSH for 24 h (mRNA levels in B, C, E) or 48 h (A, protein levels in B, C). (A) Yaku A inhibited α-MSH-induced TYR activity, velocity of dopa oxidation, in B16F0 cells. (B, C) Yaku A suppressed α-MSH-induced expression of TYR. (D) rhTYR was incubated with Yaku A for 10 min and its dopa oxidation activity was determined. Yaku A did not alter catalytic activity of rhTYR in cell-free reactions. (E) Yaku A suppressed α-MSH-induced transcription of other MITF-M-target melanogenic genes, such as TRP-1, DCT, PMEL17, Rab27A, MC1R and EDNRB. *P < 0.05 vs. α-MSH alone.

MITF-M could be up-regulated in melanocyte cultures by stimulation with α-MSH or SIK inhibitors, and its induction was suppressed by treatment with Yaku A (Figure 6D-H; Figure 8A-D). We evaluated whether Yaku A could affect the expression of MITF-M-target melanogenic genes in α-MSH-activated melanocytes. Upon exposure to α-MSH, B16F0 cells increased the velocity of dopa oxidation, which is a marker of TYR-catalyzed activity (Figure 9A). Treatment with Yaku A inhibited α-MSH-induced TYR activity in the cells (Figure 9A). Accordingly, α-MSH increased the protein and mRNA levels of TYR in HEM and B16F0 cells, which were suppressed by treatment with Yaku A (Figure 9B and C). In cell-free reactions, treatment with Yaku A did not inhibit the rhTYR-catalyzed velocity of dopa oxidation (Figure 9D). These results suggest that Yaku A could inhibit TYR activity in α-MSH-activated melanocytes by suppressing the expression of TYR but not targeting the active or allosteric site of TYR. Notably, α-MSH up-regulated the transcription of other MITF-M-target melanogenic genes, such as TRP-1, DCT, PMEL17, Rab27A, MC1R and EDNRB, in B16F0 cells, which were also suppressed by treatment with Yaku A (Figure 9E). Moreover, HEM cells up-regulated the transcription of MC1R in response to α-MSH, which was also suppressed by treatment with Yaku A (Figure S8).

**Figure 10:**
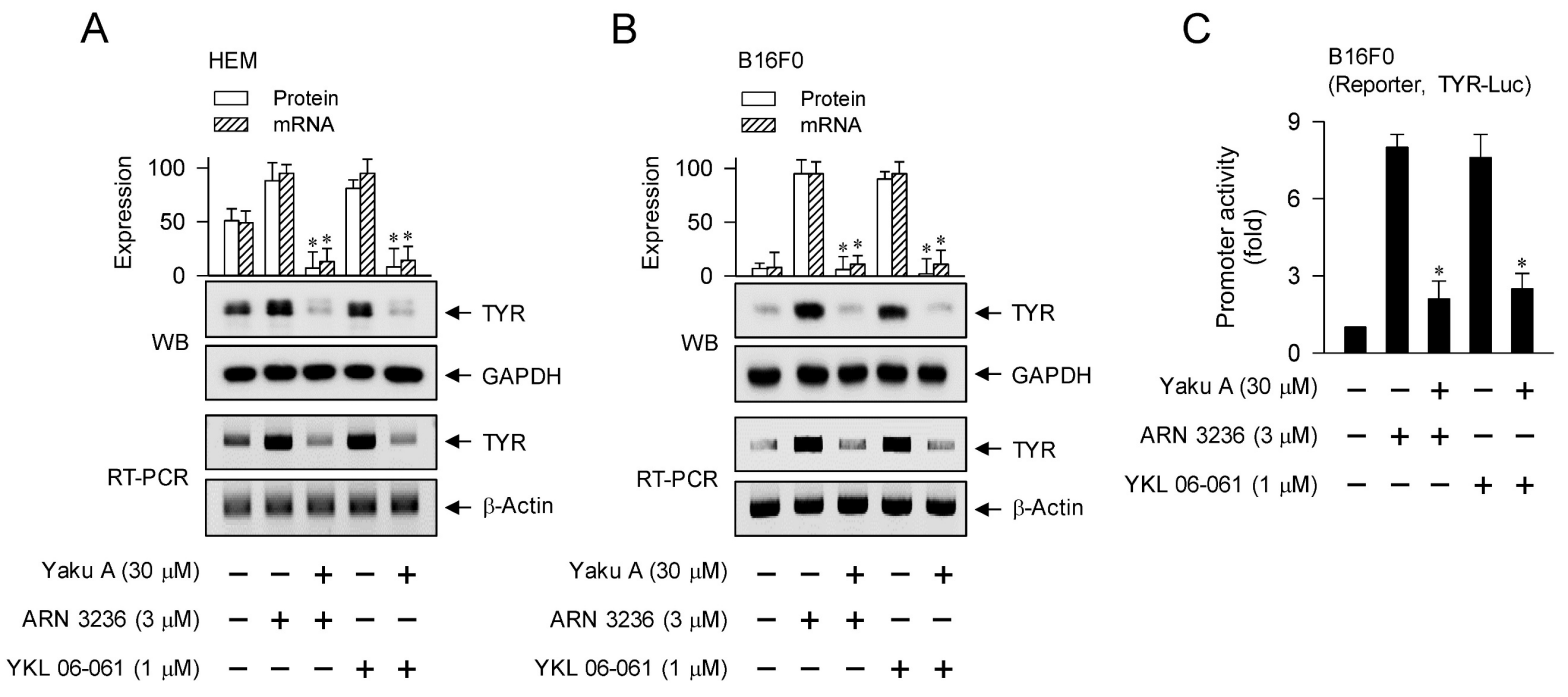

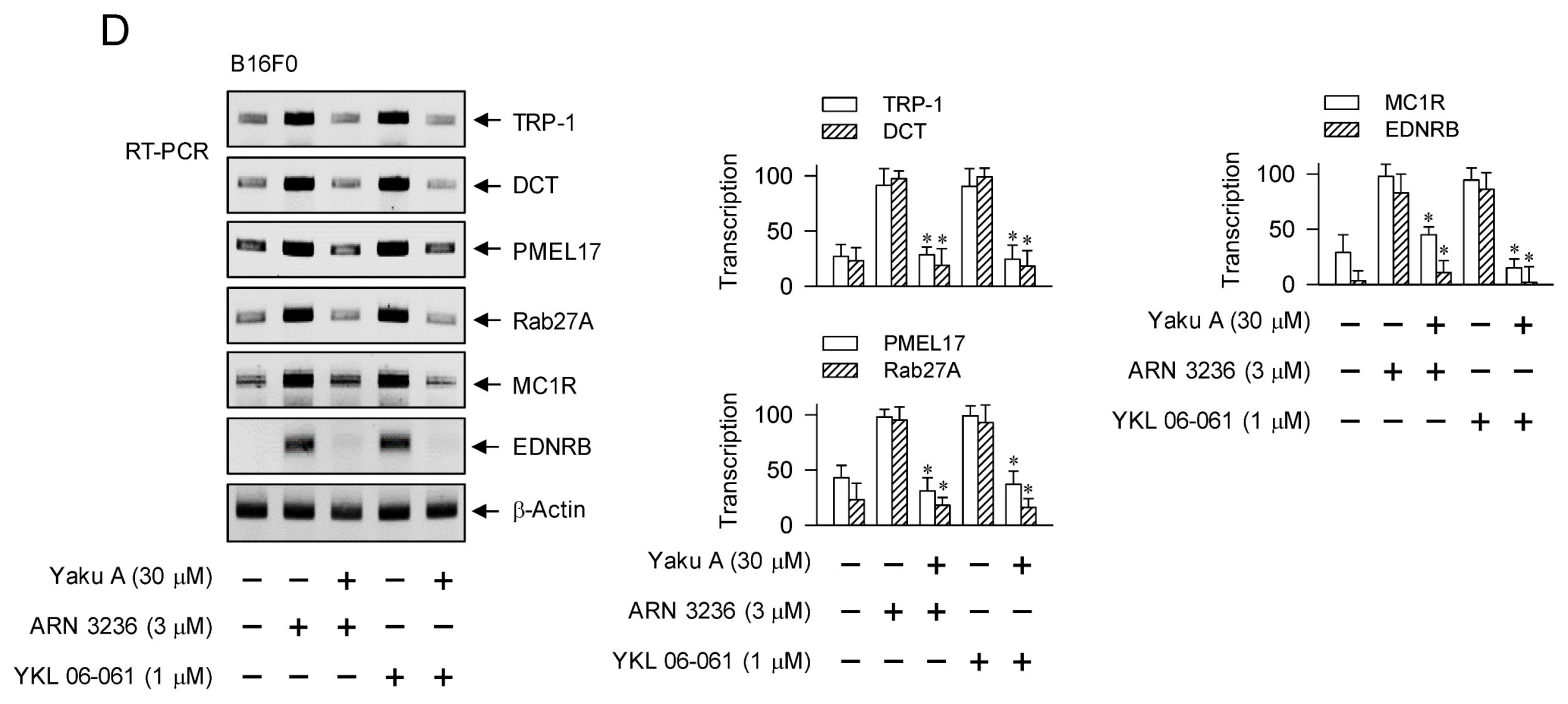
** Effect of Yaku A on the expression of MITF-M-target genes in SIK inhibitor-induced melanogenic programs.** HEM or B16F0 cells were pretreated with Yaku A for 2 h and stimulated with SIK inhibitor (ARN 3236 or YKL 06-061) for 24 h (mRNA levels in A, B, D) or 48 h (protein levels in A, B) in the presence of Yaku A. (A, B) Yaku A suppressed SIK inhibitor-induced expression of TYR. (C) B16F0 cells harboring TYR-Luc reporter construct were stimulated with SIK inhibitor in the presence of Yaku A for 20 h. Yaku A decreased SIK inhibitor-induced promoter activity of TYR. (D) Yaku A suppressed SIK inhibitor-induced transcription of other MITF-M-target melanogenic genes, such as TRP-1, DCT, PMEL17, Rab27A, MC1R and EDNRB. *P < 0.05 vs. ARN 3236 alone or YKL 06-061 alone.

We then evaluated whether Yaku A could regulate the expression of MITF-M-target melanogenic genes in SIK inhibitor-activated melanocytes. Upon exposure to SIK inhibitors, HEM and B16F0 cells up-regulated the protein and mRNA levels of TYR, which were suppressed by treatment with Yaku A (Figure 10A and B). B16F0 cells were then transfected with TYR-Luc, a luciferase reporter construct fused to the promoter region (-2236/+59) of TYR. Upon exposure to SIK inhibitors, B16F0 cells harboring TYR-Luc increased the luciferase activity, indicating the promoter activity of TYR, which was inhibited by treatment with Yaku A (Figure 10C). Furthermore, SIK inhibitors up-regulated the transcription of other MITF-M-target melanogenic genes, such as TRP-1, DCT, PMEL17, Rab27A, MC1R and EDNRB, which were suppressed by treatment with Yaku A (Figure 10D). Overall, Yaku A could suppress α-MSH- or SIK inhibitor-induced expression of MITF-M-target melanogenic genes at the promoter level.

## Discussion

In the current study, we proposed the phosphorylation circuits on CREB and CRTCs as antimelanogenic targets by elucidating the inhibitory activity and mechanism of Yaku A on facultative melanogenesis. Yaku A was identified as an antimelanognic candidate that was effective in UV-B-dependent and independent melanin production. Topical treatment with Yaku A prevented UV-B-irradiated hyperpigmentation in the dorsal skins of mice. Moreover, Yaku A inhibited melanin production in cell cultures, such as UV-B-irradiated co-culture of melanocytes and keratinocytes, and the monoculture of melanocytes stimulated with conditioned media from UV-B-irradiated keratinocytes, α-MSH, cAMP elevators (forskolin, db-cAMP and IBMX) or SIK inhibitors (ARN 3236 and YKL 06-061).

**Figure 11:**
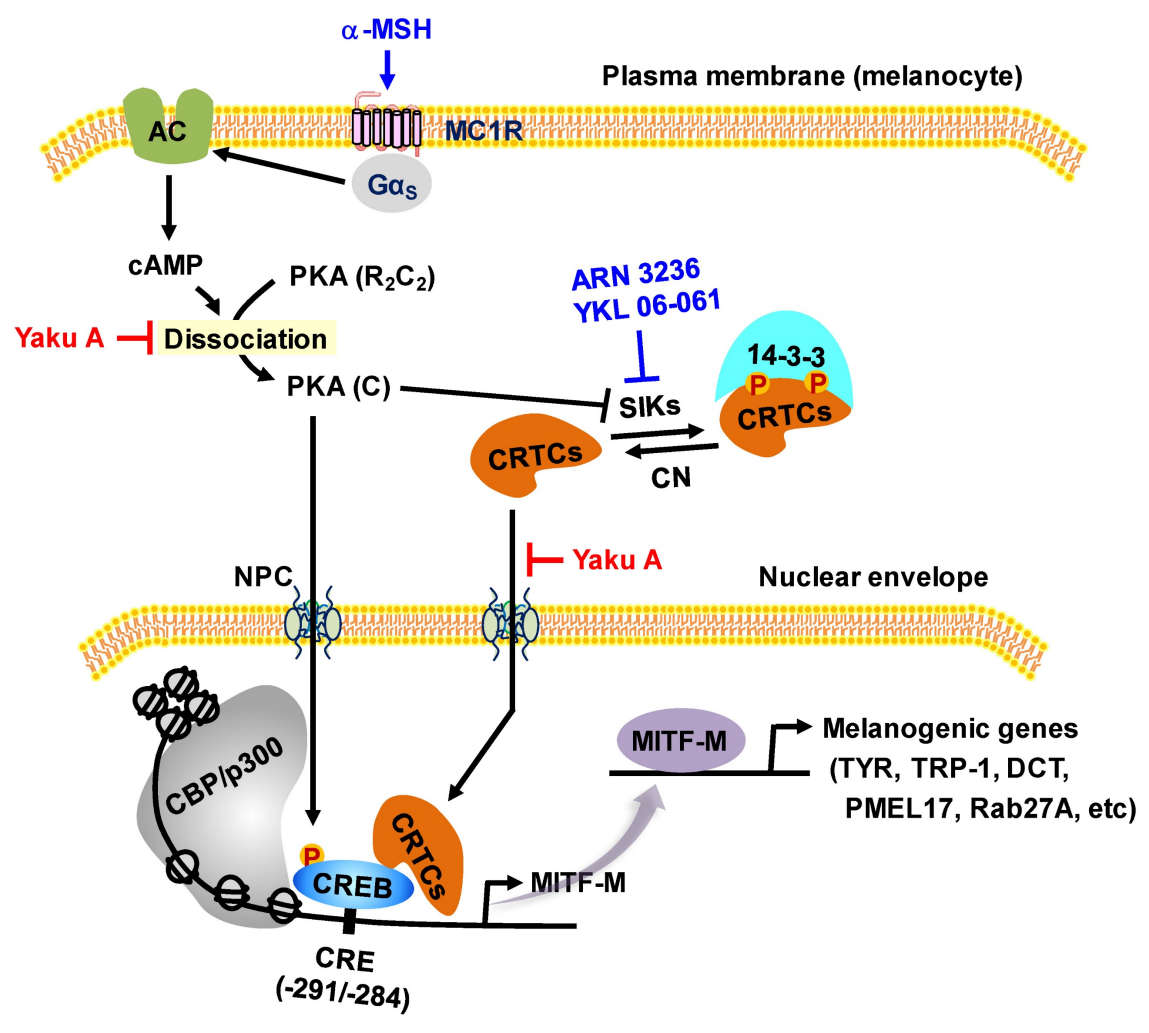
** Proposed molecular mechanism of Yaku A on antimelanogenic activity.** Yaku A suppressed the expression of MITF-M via dually targeting the i) cAMP-dependent dissociation (activation) of PKA holoenzyme, release of the catalytic subunit (C) from the regulatory subunit (R), at the upstream from PKA (C)-catalyzed phosphorylation of CREB coupled with PKA (C)-SIKs axis-mediated dephosphorylation of CRTCs in α-MSH-induced melanogenic programs and ii) nuclear import of CRTCs after SIK inhibitor-induced dephosphorylation of CRTCs in the melanogenesis. Abbreviations are AC, adenylate cyclase; CN, calcineurin; and NPC, nuclear pore complex.

The antimelanogenic mechanisms of Yaku A dually targeted the i) cAMP-dependent dissociation of PKA holoenzyme in α-MSH-induced expression of MITF-M through phosphorylation circuits on CREB and CRTCs; and ii) nuclear import of CRTCs in SIK inhibitor-induced expression of MITF-M (Figure 11). Here, we firstly report antimelanogenic chemical Yaku A with dual molecular targets, interaction between cAMP and PKA holoenzyme and translocation of CRTCs into the nucleus from the cytosol.

Yaku A primarily interrupted cAMP-dependent dissociation of the catalytic subunit PKA-Cα from the regulatory subunit PKA-RIIβ in α-MSH-activated melanocytes, as did Rp-cAMPS. The Rp-cAMPS is a cell-permeable cAMP antagonist, blocks the dissociation of PKA-Cα from PKA-RIIβ, PKA-RIα or PKA-RIβ, and inhibits melanin production in α-MSH-activated and cAMP-elevated melanocyte cultures 36. Yaku A mimicked Rp-cAMPS in the antimelanogenic mechanism, even though chemical structure of Yaku A is not analogous to those of Rp-cAMPS and agonist cAMP. Antimelanogenic chemicals such as bisabolangelone and diphenylmethylene hydrazinecarbothiomide also blocks cAMP-dependent activation of PKA via targeting the dissociation of catalytic subunits from the holoenzyme 36, 37. The PKA holoenzyme comprises a heterotetrameric molecule with two catalytic and two regulatory subunits 38. There are three isoforms of the catalytic subunits (PKA-Cα, Cβ and Cγ) and four isoforms of the regulatory subunits (PKA-RIα, RIβ, RIIα and RIIβ) 38. The regulatory isoforms of PKA share the conserved and well-defined structures; an N-terminal dimerization domain, an interconnecting linker that docks to the active site cleft in the catalytic subunit, and a C-terminal domain that encompasses two tandem cAMP-binding sites 38. The binding of cAMP to regulatory subunits in the inactive PKA holoenzyme causes a conformational change and results in the release of catalytic subunits, thus activating the kinase activity of PKA that phosphorylates protein substrates, including CREB, SIKs and AMPK 39.

Yaku A directly blocked nuclear import of all isoforms of CRTCs in SIK inhibitor-activated melanocytes. Antimelanogenic chemical diacetylcaffeic acid cyclohexyl ester inhibits nuclear entry of CRTC1 in α-MSH-, endothelin 1- or SIK inhibitor-activated melanocyte cultures, and its topical treatment protects the skin from UV-B-irradiated hyperpigmentation in mice 31. Chemical structure of Yaku A is not related to that of diacetylcaffeic acid cyclohexyl ester. Although nuclear-cytoplasmic shutting of CRTCs across nuclear pore complex remains to be defined at molecular level, nuclear localization sequences are identified in each isoform of CRTCs 40, 41. KPNA1 (also called Impα5) escorts CRTC1 from the cytosol to the nucleus, but siRNAs targeting KPNA2 (Impα1) and KPNA4 (Impα5) do not alter the nuclear import of CRTC1 31.

α-MSH-induced melanogenic programs increased PKA-catalyzed phosphorylation of CREB at the S133 residue. CREB was predominantly localized in the nucleus of melanocytes, and its phosphorylation occurred in the nucleus upon exposure to α-MSH or cAMP elevators but not SIK inhibitors. CREB contains the central transactivation domain (TAD) and C-terminal basic Leu zipper (bZIP) domain 14. The phosphorylation of CREB at the S133 residue in the TAD is critical to recruit CBP/p300 that participate in the epigenetic regulation of chromatin remodeling to become transcriptionally active 12, 13. The bZIP domain in CREB has several subdomains, such as those responsible for nuclear localization, interaction with CRTCs, and binding to DNA 14.

The dephosphorylation of CRTCs was increased in α-MSH- or SIK inhibitor-induced melanogenic programs. Phosphorylated CRTCs were predominantly localized in the cytosol of melanocytes. Upon exposure to SIK inhibitors, CRTCs were dephosphorylated and translocated from the cytosol to the nucleus. Three isoforms of CRTCs share the N-terminal CREB-binding domain, central regulatory domain, Ser/Gln-rich splicing domain and C-terminal TAD 14. Kinase activities in SIKs or AMPK phosphorylate the conserved Ser residues in the regulatory domain of each CRTC (S64, S151, S167 and S245 residues in CRTC1; S70, S171, S274, S306 and S368 residues in CRTC2; and S62, S162, S273, S329, and S370 residues in CRTC3), which sequester CRTCs in the cytosol by tethering with 14-3-3 protein 41, 42. JNK activity also phosphorylates CRTC3, which blocks nuclear entry of CRTC3 and inhibits melanogenesis via interfering with CRTC3-dependent MITF-M expression in melanocyte cultures 43. In contrast, ERK1/2-catalyzed phosphorylation of CRTC3 at the S391 residue increases its interaction with calcineurin, hydrolyzing the phosphor groups of CRTC3 at the binding sites with 14-3-3 protein 44.

CRTCs/CREB mainly regulate a broad network that senses hormonal and metabolic homeostasis signals 14, which might be impaired in the whole-body knock-out (KO) or overexpression of each isoform of CRTCs. Mice with a KO of CRTC3 but not CRTC1 or CRTC2 decrease fur pigmentation, and CRTC3-KO mice down-regulate the transcription of OCA2, but not those of other melanogenic genes such as encoding MITF-M and TYR 33. In another, CRTC3-KO mice are paler in coat and skin color due to a reduced deposition of melanin pigments, and down-regulate the transcription of MITF-M, TYR and OCA2 32. Overexpression of CRTC1, CRTC2 or CRTC3 was capable of inducing the enhanced melanogenesis, but that of dominant-negative CRTC1 inhibited melanin production in UV-B-irradiated melanocyte cultures 30. Antimelanogenic chemical diacetylcaffeic acid cyclohexyl ester suppresses the transcription of MITF-M via blocking nuclear entry of CRTC1 in α-MSH-activated melanocyte cultures, and its topical treatment mitigates hyperpigmentation in UV-B-irradiated skins of brownish guinea pigs 31. Adiponectin, an AMPK activator, increases the phosphorylation of CRTC2 and CRTC3, down-regulates the expression of MITF-M, and decreases melanin contents in Mel-Ab cultures, and its mRNA levels are lower in the lesional skin of melasma patients compared with non-lesional skin 45.

As shown in Figure 1, this study suggests that α-MSH-induced and cAMP-dependent melanogenic programs in the cultures of B16F0 cells could switch on the axis of PKA-SIKs but not that of PKA-AMPK in the dephosphorylation of CRTCs. SIK inhibitors rather than AMPK inhibitors stimulated melanin production in the cultures of HEM or B16F0 cells, wherein SIK inhibitors increased the dephosphorylation of CRTCs but bypassed the phosphorylation of CREB in the expression of MITF-M.

Three isoforms of SIKs (SIK1, SIK2, and SIK3) share the N-terminal kinase domain, ubiquitin-associated domain, and the C-terminal tail 10. Kinase activities of SIKs are negatively regulated by PKA-catalyzed phosphorylation of the C-terminal domain in each SIK (T473 and S575 residues in SIK1; S343, S358, T484 and S587 residues in SIK2; and T469, S551 and S626 residues in SIK3) 10. SIK2-KO promotes melanogenic programs in mice regardless of the presence or absence of melanogenic stimuli; these include the pigmentation switch from pheomelanin to eumelanin in A^y^/a mice that show a yellow coat due to the inactivation of MC1R by overexpression of the agouti protein 30.

In the current study, inhibition of AMPK activity by pharmacological agents (BAY 3827 and dorsomorphin) at 1-30 μM could not stimulate melanin production in HEM or B16F0 cells, suggesting a common phenomenon of melanocytes. In contrast, AMPK activators (adiponectin and acadesine) at 5-100 μM stimulate the phosphorylation of CRTC2 and CRTC3, suppresses the expression of melanogenic genes, and decreases melanin contents in Mel-Ab melanocytes 45. Further study remains to conclude whether AMPK activity would be important or dispensable in the melanogenic programs.

## Conclusions

Taken together, we propose that the targeting of phosphorylation circuits on CREB and CRTCs in the expression of MITF-M is a suitable strategy for the treatment of pigmentary disorders in the skin.

## Supplementary Material

Supplementary appendix, figures and table.
