# Supplementary material for: Targeting phosphorylation circuits on CREB and CRTCs as the strategy to prevent acquired skin hyperpigmentation
Source: Int J Biol Sci. 2024 Jan 1;20(1):312–30. doi: 10.7150/ijbs.86536 (PMC10750286; doi:10.7150/ijbs.86536)
Supplement: Supplementary file 1 — Supplementary appendix, figures and table. [file ijbsv20p0312s1.pdf]

Supplementary materials

Targeting phosphorylation circuits on CREB and CRTCs as the strategy to prevent acquired skin hyperpigmentation

Song-Hee Kim[1,*], Changseon Na[1,*], Cheng-Yong Yun[1,2], Jun Gu Kim[1], Seung Tae Baek[2], Hyun Jin An[3], Jae Duk Lee[3], Seung Wha Lee[1], Jae-Kyung Jung[1], Bang Yeon Hwang[1], Sang-Bae Han[1✉], and Youngoo Kim[1✉]

[1]College of Pharmacy, Chungbuk National University, Cheongju 28160, Korea; [2]R&D Center, The Skin's Co. Ltd, Jecheon 27116, Korea; [3]R&D Center, Yeomyung Biochem Co. Ltd, Cheongju 28172, Korea

---

Contents

Appendix S1.

Table S1.

Figures S1 to S8.

Appendix S1. Supplementary methodology: isolation of Yaku A from *Alpinia oxyphylla*.

General experimental procedures. UV spectra were recorded on a JASCO UV-550 spectrophotometer, and IR spectra were measured on a JASCO FT-IR 4100 spectrometer. NMR spectra were recorded on a Bruker AVANCE 400 MHz, spectrometer using $CDCl_3$ as solvent. HRESIMS were obtained with Orbitrap Exploris 120 mass spectrometer. Column chromatography was performed on silica gel (Merck, 70-230 mesh) and Sephadex LH-20 (25-100 μm, Pharmacia). HPLC analysis was performed using Waters HPLC system equipped with two Waters 515 pumps with a 2996 photodiode-array detector using a YMC J'sphere column (4 μm, 150 x 4.6 mm, i.d. with flow rate 1.0 mL/min and 4 μm, 150 × 20 mm, i.d. with flow rate 6.0 mL/min). TLC was performed using precoated silica gel 60 F_{254} (0.25 mm, Merck) plates, and spots were detected by a 10% vanillin-H_2SO_4 in water spray reagent.

Plant material. The dried fruits of *A. oxyphylla* were purchased from Kyungdong herbal market in Seoul, Korea, in October 2019. A voucher specimen (CBNU-2019-10-AO) was authenticated by B.Y.H. and deposited at the Herbarium of the College of Pharmacy, Chungbuk National University, Korea.

Extraction and isolation. The dried fruits of *A. oxyphylla* (1.0 kg) were extracted with MeOH (3 x 5 L) at room temperature for 3 days. The extract was evaporated under reduced pressure, and the residue (150 g) was suspended in water and partitioned successively with *n*-hexane (2 x 2 L), CH_2Cl_2 (2 x 2 L), and EtOAc (2 x 2 L). The CH_2Cl_2-soluble fraction (25.0 g) was chromatographed on a silica gel column and eluted with *n*-hexane-EtOAc gradient system (10:1 to 0:1) to give seven fractions (AOC1 – AOC7). AOC4 (3.0 g) was purified by Sephadex LH-20 gel with *n*-hexane: CH_2Cl_2: MeOH = 5: 5: 1 isocratic solvent system to remove dark color pigment, and afford yakuchinone A (Yaku A, 500 mg).

Table S1. Nucleotide sequences of RT-PCR primers.

Target	Nucleotide sequence		Amplicon
DCT (B16F0)	Forward	5’-ACAGACGACTGGCTTGGAGCAGCAA-3’	517 bp
	Reverse	5’-5’-ACATTCGGTTGTGACCAATGGGTGC-3’	
EDNRB (B16F0)	Forward	5’-TCCTGGACCAGAGGTTCCAA -3’	333 bp
	Reverse	5’-TGCGAGCAACTTGTAGGTGT-3’	
MC1R (B16F0)	Forward	5'-TGAGTCTGGTGGAGAATGTG-3'	528 bp
	Reverse	5'-TTTTGTGGAGCTGGGCAATGC-3’	
MC1R (HEM)	Forward	5'-TCGCCGTGGACCGCTACATC -3'	119 bp
	Reverse	5'-GCGTG CTGAA GACGA CACTG-3’	
MITF-M (B16F0)	Forward	5’-CTAGAATACAGTCACTACCAG-3’	404 bp
	Reverse	5’-CCATCAAGCCCAAAATTTCTT-3’	
MITF-M (B16F0, HRM2)	Forward	5’-GACTAAGTGGTCTGCGGTGT-3’	153 bp
	Reverse	5’-TGCTTTACCTGGTGCCTCTG-3’	
MITF-M (HEM)	Forward	5'-TCTACCGTCTCTCACTGGATTG-3'	142 bp
	Reverse	5'-GCTTTACCTGCTGCCGTTGG-3’	
PMEL17 (B16F0)	Forward	5'-ATCAATGGGAGCCAGGTGTG-3'	479 bp
	Reverse	5'-AGGGTCCCAGTACCATCTCC-3’	
Rab27A (B16F0)	Forward	5'-GGGCAGGAGAGGTTTCGTAG-3'	508 bp
	Reverse	5'-CTTGGTCTCTACAGCGGAGC-3’	
TRP-1 (B16F0)	Forward	5'-GATATGGCGAAGCGCACAACTCACC-3'	536 bp
	Reverse	5'-AGACGCTGCACTGCTGGTCTCCCTA-3’	
TYR (B16F0)	Forward	5’-TACAGTCACTACCAGGTGCAG-3’	1211 bp
	Reverse	5’-CCATCAAGCCCAAAATTTCTT-3’	

TYR (B16F0, HRM2)	Forward	5’-ACACACTGGAAGTATTTTTGAACA-3’	254 bp
	Reverse	5’-GCCAGATACGACTGGCTTGT-3’	
TYR (HEM)	Forward	5'-TACTGGGATAGCGGATGCCT-3'	325 bp
	Reverse	5'-AGAGTCTGGGTCTGAATCTTGT-3’	
β-Actin (B16F0)	Forward	5’-TGGAATCCTGTGGCATCCATGAAAC-3’	349 bp
	Reverse	5’-TAAAACGCAGCTCAGTAACAGTCCG-3’	
β-Actin (HEM)	Forward	5'-GGACTTCGAGCAAGAGATGG-3'	234 bp
	Reverse	5'-AGCACTGTGTTGGCGTACAG-3‘	

Abbreviation: bp, base pairs.

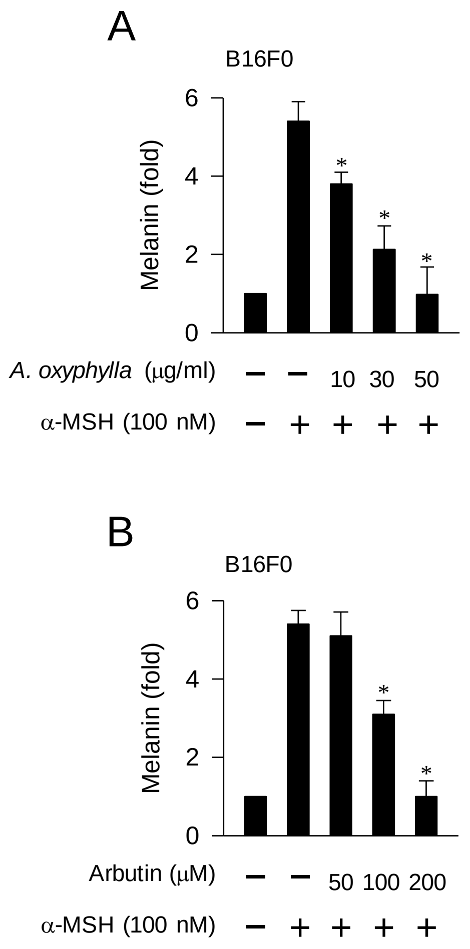

Figure S1. Inhibitory effect of *Alpinia oxyphylla* extract on melanin production in α-MSH-activated B16F0 cells. The cells were stimulated with α-MSH in the presence of *A. oxyphylla* extract (A) or arbutin (B) for 72 h. *A. oxyphylla* extract and arbutin inhibited α-MSH-induced melanin production in the cultures of B16F0 cells. *$P < 0.05$ vs. α-MSH alone.

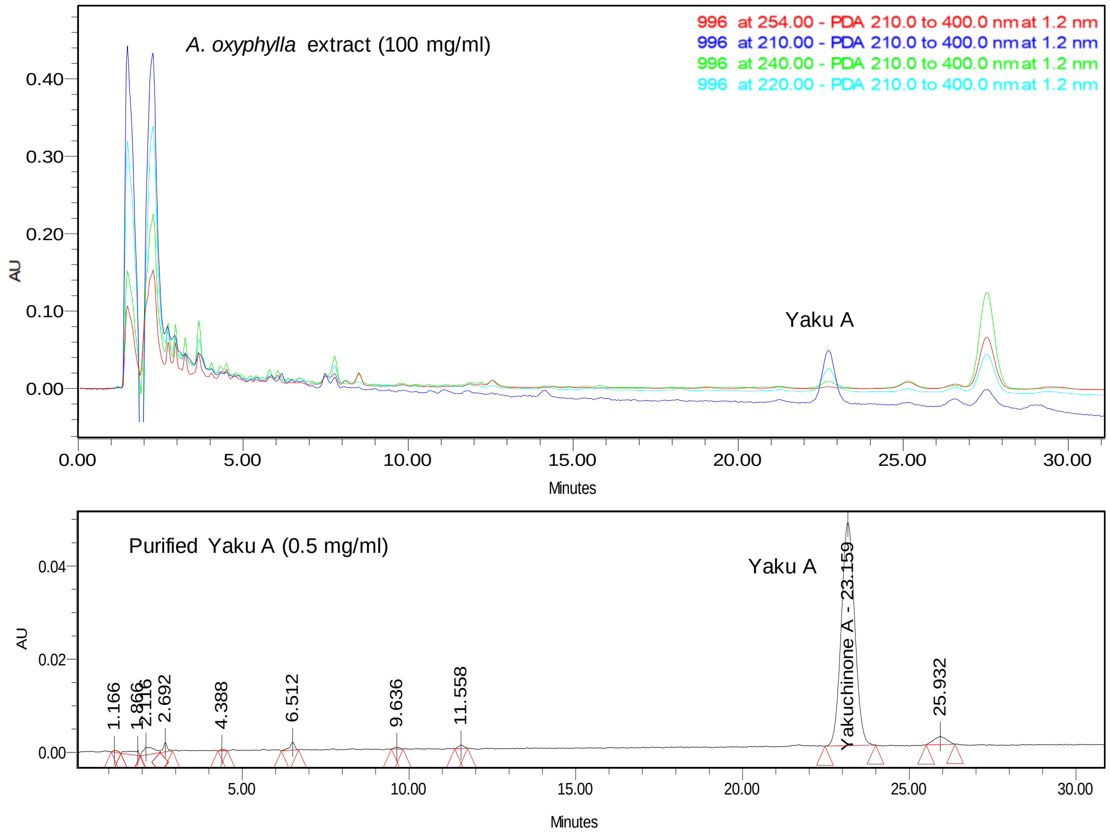

Figure S2. HPLC-DAD chromatograms of *Alpinia oxyphylla* extract and purified Yaku A. *A. oxyphlla* extract (upper) or purified Yaku A (lower) was analyzed by Waters Alliance 2695 HPLC system with 2996 photodiode array detector, YMC J'sphere ODS-H80 column (4 µm, 150 x 250 mm, i.d.). Isocratic solvent system of CH_3CN and 0.1% formic acid in water (50:50, v/v, flow rate = 1.0 ml/min, 30 min) was used for the seperation.

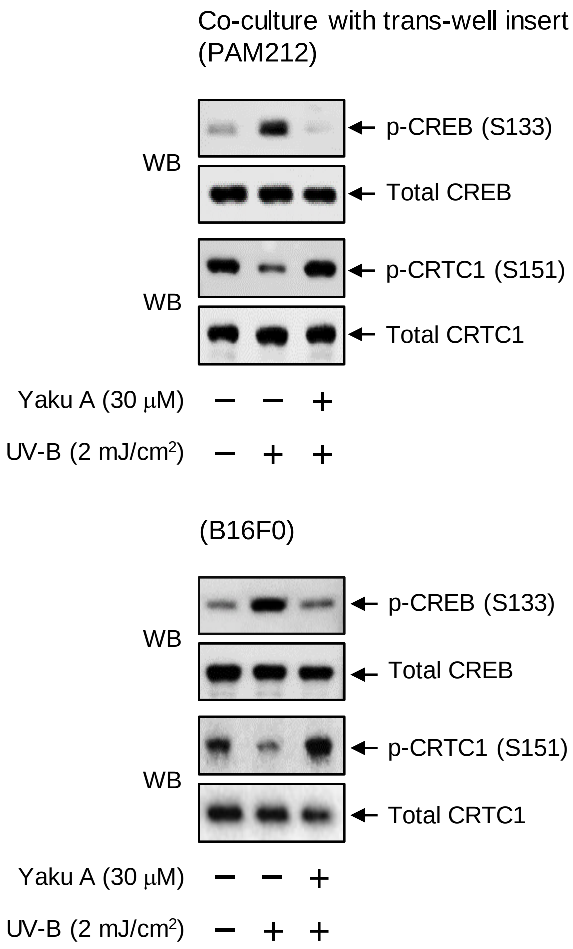

Figure S3. Effects of Yaku A on CREB phosphorylation and CRTC1 dephosphorylation in UV-B-irradiated co-culture of PAM212 keratinocyte and B16F0 melanoma cells. Co-culture with trans-well insert between PAM212 and B16F0 cells was pretreated with Yaku A for 2 h. After stimulation with a single irradiation of UV-B, the co-culture was incubated in the presence of Yaku A for another 30 min. Treatment with Yaku A inhibited UV-B-irradiated phosphorylation of CREB at the S133 residue and the dephosphorylation of CRTC1 at the S151 residue in PAM212 cells (upper) as well as those in B16F0 cells (lower).

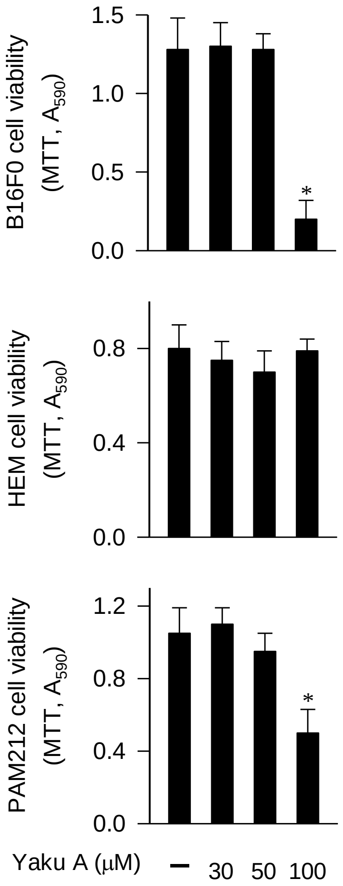

Figure S4. Effect of Yaku A on cell viability. B16F0, HEM or PAM212 cells were incubated with Yaku A for 72 h, and reacted with MTT for another 30 min. Yaku A at 30-50 μM did not alter cell viability. **P* < 0.05 vs. medium alone.

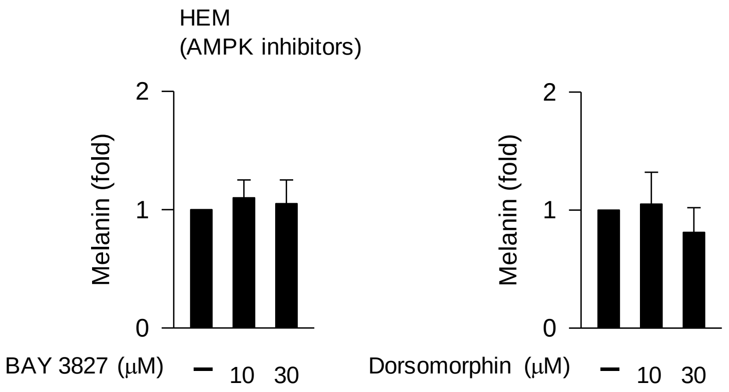

Figure S5. Effects of AMPK inhibitors on melanin production in HEM cells. The cells were incubated with AMPK inhibitor (BAY 3827 or dorsomorphin) for 72 h. AMPK inhibitors did not alter melanin production in the cultures of HEM cells.

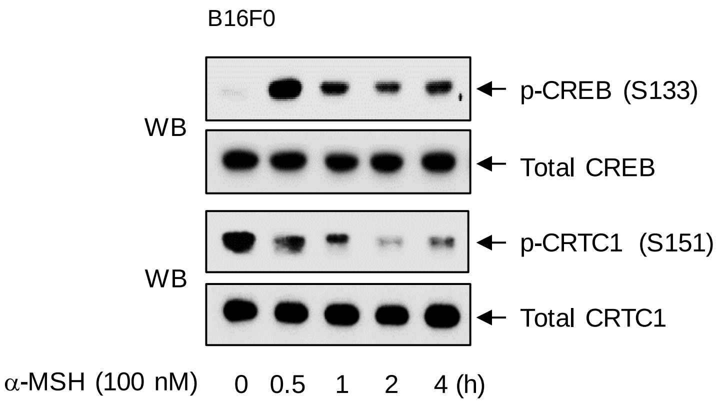

Figure S6. Time course study of α-MSH-induced CREB phosphorylation and CRTC1 dephosphorylation in B16F0 cells. The cells were stimulated with α-MSH for indicated time points, and their protein extracts were subjected to Western blot (WB) analysis. α-MSH-induced phosphorylation of CREB at the S133 residue was peaked at 30 min and returned to low levels of p-CREB from 1 h to 4 h. α-MSH dephosphorylated CRTC1 at the S151 residue within 30 min, and low levels of p-CRTC1 were sustained until 4 h.

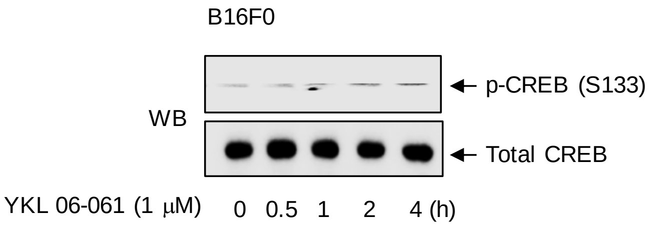

Figure S7. Time course study of YKL 06-061-induced CREB phosphorylation in B16F0 cell. The cells were stimulated with YKL 06-061 for indicated time points, and their protein extracts were subjected to Western blot (WB) analysis. YKL 06-061 did not phosphorylate CREB at the S133 residue during 4 h.

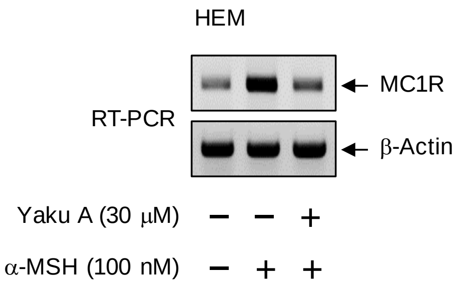

Figure S8. Effect of Yaku A on α-MSH-induced transcription of MC1R in HEM cells. The cells were pretreated with Yaku A for 2 h and stimulated with α-MSH for 24 h in the presence of Yaku A. Total RNAs were subjected to RT-PCR analysis. The mRNA levels of MC1R, a target gene of MITF-M, were up-regulated in the cultures of α-MSH-activated HEM cells, which was suppressed by treatment with Yaku A.
